# Strategic foresight provides insight into the resilience of climate-neutral farming transitions in a volatile world

**DOI:** 10.1038/s44264-026-00185-2

**Published:** 2026-08-31

**Authors:** David Styles, Daniel Henn, Colm Duffy, Kevin Black, Andres Martinez-Arce

**Affiliations:** 1 School of Biological & Chemical Sciences and Ryan Institute, University of Galway, Galway, Ireland; 2 Forest, Environmental Research & Services (FERS) Ltd, Navan, Ireland

**Keywords:** Climate sciences, Ecology, Ecology, Environmental sciences, Environmental social sciences

## Abstract

Farmers manage almost 5 billion hectares of land globally, deliver food security and underpin cultural and economic life in rural areas. Farming policies typically prioritise stability and incremental change to mitigate risk. Yet scientific evidence points to a need for transformative change across the land sector to effectively tackle the climate and biodiversity crises. Meanwhile, climate and geopolitical volatility underscore the need for resilient farm systems. Reconciling multifaceted objectives within long-term strategic policymaking for farming and land use is a major challenge, requiring multi-dimensional evidence. Using Ireland’s land sector as an example, we employ strategic foresight combined with quantitative back-casting to explore alternative climate-neutral farming pathways under different future contexts. Bioeconomy and protein diversification pathways score better across sustainability, resilience and risk indicators compared with business as usual (BAU) under uncertain futures represented by shared socioeconomic pathways. These findings help to situate the important, tangible risks associated with climate-neutral transitions against multifaceted risks facing farming stakeholders under BAU.

## Introduction

Farmers manage almost 5 billion hectares (38%) of global land area^1^ to produce marketable food, bioenergy and biomaterials, alongside the delivery of multiple non-marketed ecosystem services. Globally, agriculture, forestry and other land use (AFOLU) contributes over 11.9 ± 4.4 Gt CO₂e yr⁻¹ (21%) of net anthropogenic greenhouse gas (GHG) emissions^2^, 44 Tg N yr⁻¹ of ammonia emissions^3^ and 0.6 Tg of phosphorus losses to water^4^, contributing to exceedance of multiple planetary boundaries^5^. Meanwhile, the AFOLU sector is highly vulnerable to climate change impacts^2^, with diminishing resilience linked to resource depletion, soil degradation and biodiversity decline^6–8^. Climate tipping points such as a collapse of the Atlantic Meridional Overturning Circulation could force dramatic (unplanned) transformation of European farming under high GHG emission scenarios^9^. Policies to halt biodiversity decline, such as the European Union’s Nature Restoration Regulation^10^, will require large areas of land to be spared from conventional agricultural production—potentially driving a combination of land sparing and land sharing at different scales^11^. Global modelling implies that (planned) transformative change (large shifts in farming practises, diets and land management) will be necessary to deliver food security within planetary boundaries^12^, and to deliver negative emissions and feedstocks for the bioeconomy to curtail climate change^13^. Given the scale of the climate and biodiversity crises, and constrained rates of change in land use and climate responses, there is an urgent need for long-term strategic land use planning. Juxtaposed with this scientific evidence on the imperative for change to tackle sustainability and resilience challenges are the socio-economic and cultural factors that “lock-in” current practises. Agriculture accounts for just 4% of global gross domestic product^14^, yet makes a vital contribution to rural employment and cultural identity, as well as delivering essential products and services. Farmers have relatively little market power within global industrialised agrifood value chains^15^. Recent attempts to restructure farming in line with sustainability objectives have met with political resistance and civil unrest^16^, and are often framed as “bad for farmers” by agri-food sector lobby groups and farming media^17^. Thus, implementing AFOLU transitions is perceived by policymakers and farmers as high risk. Narrow framing around short-term interests^17^ may neglect longer-term risks associated with the first- and second-order effects of climate change, including increasing economic instability and uncertainty^18^. The confluence of complex challenges facing the AFOLU sector globally may be defined as a “wicked problem”^19^. There is a need to move beyond single-issue climate mitigation modelling of AFOLU measures towards more holistic evaluation of co-benefits, trade-offs and risks associated with alternative AFOLU development pathways in order to inform strategic policymaking for farming.

Ireland is a microcosm of this global AFOLU challenge, with a large export-oriented agrifood sector responsible for 37% of national GHG emissions^20^, underpinned by 133,000 small farms, many of which rely on subsidies to continue operating^21^. Ninety percent of milk and beef produced is exported, so the sector is highly dependent on the continuation of open markets and international demand, as well as subsidies. Farmers are concerned about climate change impacts, yet also worry about excessive regulation^22^ and there is strong resistance to diversification out of livestock farming^23^. Achieving “climate neutrality” by 2050 is a legally binding target for Ireland’s economy^24^, though the definition of national climate neutrality remains under debate^25^. The specific questions this study seeks to answer are: (i) how do indicative climate-neutral AFOLU transitions compare with business as usual (BAU) in terms of wider societal demands from land? (ii) how do near-term risks associated with implementing such transitions compare with future risks facing AFOLU stakeholders under different plausible futures in a changing world? To answer these questions, we apply two powerful approaches to inform strategic policymaking in AFOLU.

Back-casting is a modelling approach that works back from fixed constraints or objectives, and has been used to identify AFOLU configurations that comply with net zero GHG emissions^26,27^. Here, we combine back-casting and stakeholder consultation to develop AFOLU pathways that comply with two definitions of national climate neutrality by 2050: (i) net zero CO_2_e, emissions; (ii) a split gas 32% reduction target for methane with net zero emissions of long-lived gases. Dairy and beef cattle sectors, as dominant GHG emitters, were constrained to comply with aforementioned climate targets, dependent on, *inter alia*, abatement technologies employed and the scale of afforestation and technical carbon dioxide removal (CDR) achieved.

**Fig. 1:**
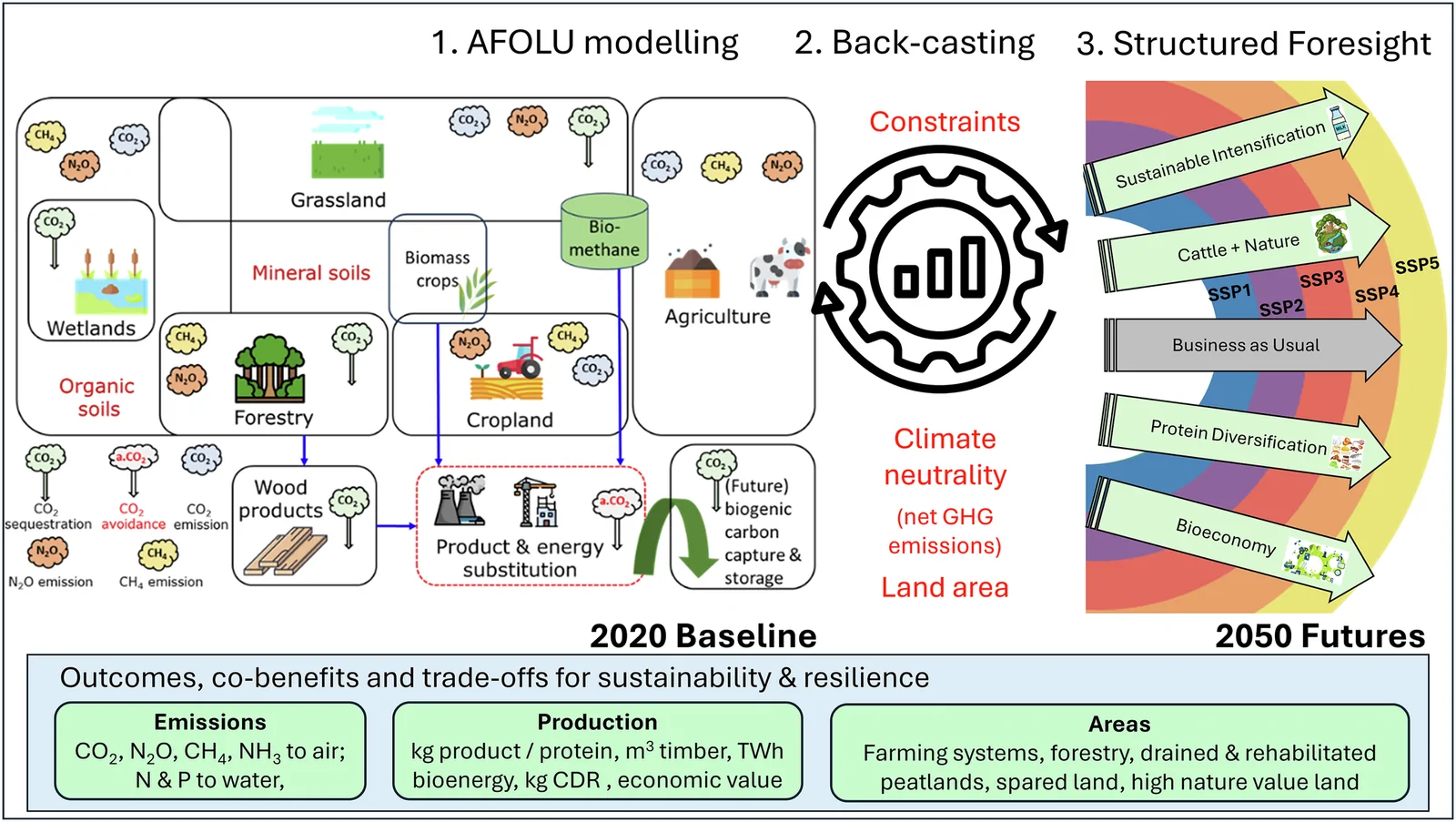
Conceptual representation of business as usual and four alternative agriculture and land use pathways modelled in this study. The GOBLIN back-casting model was used to identify configurations that operate within specified definitions of “climate neutrality” by 2050 for four of those pathways (delineating “climate-neutral transitions”). Pathways intersect with uncertain futures, described using the Shared Socioeconomic Pathway (SSP) framework.

Strategic foresight is a systems approach to explore plausible futures, providing evidence on transformative change not well represented in the economic models often used to inform policy^18,28^. Here, foresight is employed to develop four AFOLU transition pathways, and to evaluate those pathways alongside BAU for wider sustainability and resilience. The transition pathways elucidate different land use priorities suited to Ireland’s agroecological conditions and farming sector, including: “Sustainable Intensification” (SI) to produce as much cattle protein as possible within land or climate constraints; Cattle + Nature (CN) involving low stocking densities on low-input, biodiverse grasslands (agroecological approach); Protein Diversification (PD) involving more pig, poultry and protein crop production alongside SI and grass-protein biorefineries^29^; Bioeconomy (BE) based on PD plus 10% of grassland planted with short rotation coppice willow as a bioenergy feedstock (Fig. 1). GHG emissions and other environmental impacts are derived from GOBLIN model^30^ biophysical outputs at national scale. Resilience is considered in terms of risks associated with pertinent biophysical, socio-economic and geopolitical factors that could manifest under the different SSP futures—representing the uncertain contexts in which AFOLU policies will play out over coming decades. Crucially, given rate-constrained changes in AFOLU, pathway choice is considered to be independent of SSP future contexts—the interactions are critical (Fig. 1). A semi-quantitative scoring system is elaborated in Table S5.

## Results

### Climate change mitigation

**Fig. 2:**
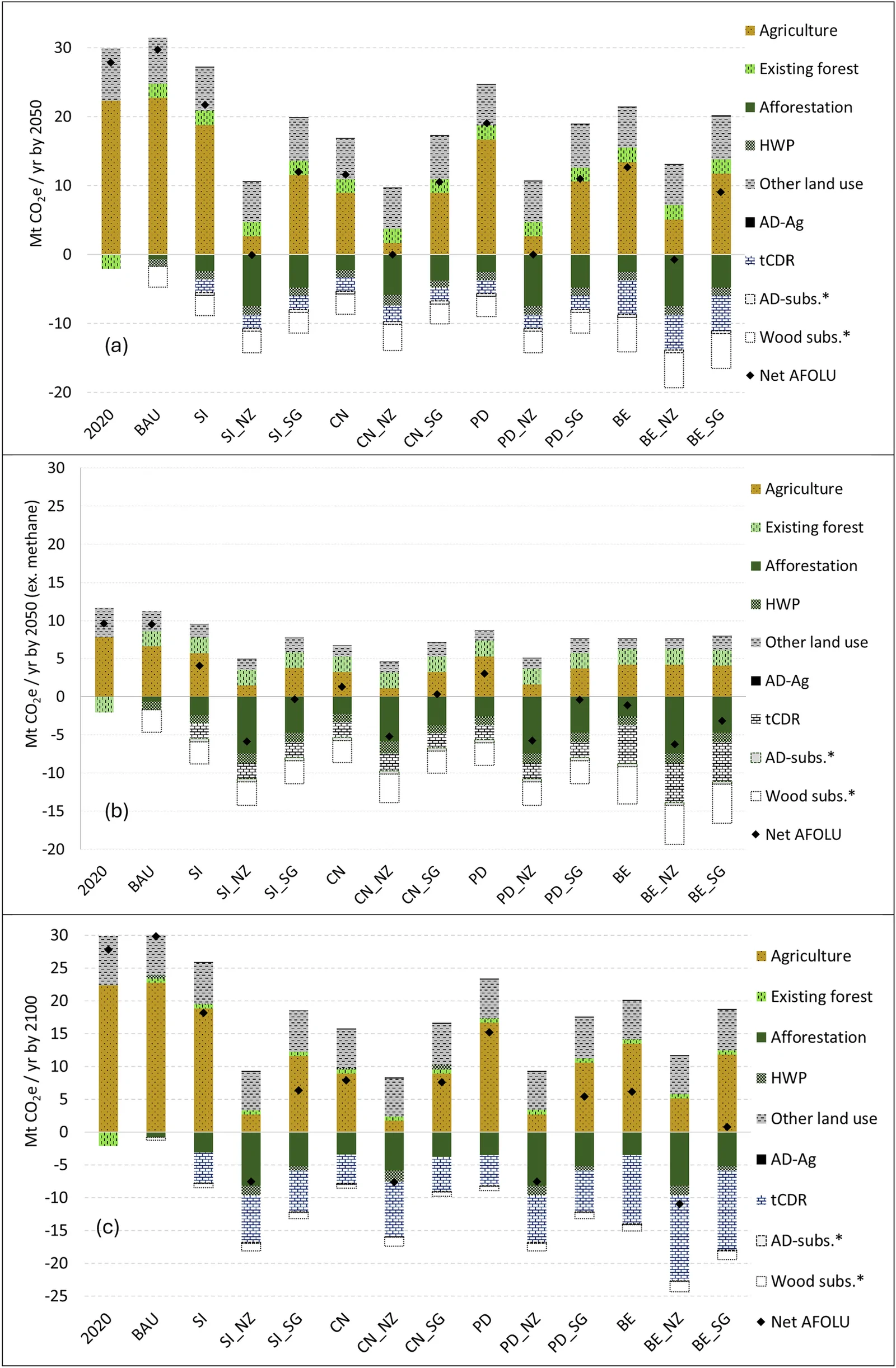
Greenhouse emissions and removals by source in 2050 and 2100. Stacked column charts displaying greenhouse gas emission balance for business as usual and variations of the four alternative agriculture, forestry and other land use (AFOLU) pathways—Sustainable Intensification, Cattle + Nature, Protein Diversification and Bioeconomy. Alternative pathway derivatives are: (i) are not constrained by any climate target, or; (ii) comply with a net zero (NZ) or (iii) a split gas (SG) greenhouse gas emission target by 2050. Panel (**a**) displays all-gas CO_2_e emissions in 2050; panel (**b**) displays only long-lived CO_2_ and nitrous oxide emissions in 2050 (excluding short-lived methane which is set a separate target in the SG pathways); panel (**c**) displays all-gas CO_2_e emissions by 2100. Harvested wood products (HWP) and technical carbon dioxide removal (tCDR) are counted in the AFOLU emissions balance; downstream substitution effects are shown for context only, and do not count towards AFOLU climate neutrality calculations in back-casting.

Figure 2 displays the GHG flux profile for Ireland’s AFOLU sector in 2020 and in 2050 for the 13 scenarios explored—BAU and four transition pathways, each of which is: (i) non-GHG-constrained; (ii) net zero CO_2_e emission constrained, or; (iii) split-gas emission (or land) constrained. Full time series are available in the supplementary information data file (S1 to S5). Relative to 2020, BAU results in an increase in emissions to 29.7 Mt CO_2_e. Other non-emissions-constrained scenarios would result in annual net emissions of between 11.6 (CN) and 21.7 (SI) Mt CO_2_e (Fig. 2a). Livestock numbers in the CN scenario are constrained by available land area because of a shift to more extensive cattle systems that support co-delivery of ecosystem services (ES), whilst high productivity cattle and high stocking rates combined with ambitious deployment of “frontier” abatement technologies (e.g. enteric methane inhibitors, anaerobic digestion) in the SI scenario moderate livestock reductions (Table 3). Conversely, achieving net zero emissions in AFOLU requires a 90% reduction in agricultural emissions, such that there is no emissions space for cattle production in the net zero CN pathway after emissions from cropping systems, pigs, poultry and sheep are accounted for (Table S1). Acknowledging the distinct warming dynamics of methane and a non-zero target for emissions of this gas globally to achieve climate stabilisation^31^, we focus on split gas climate neutral transition pathways henceforth. These pathways result in net GHG emissions of 9 (BE) to 12 (SI) Mt CO_2_e by 2050 (Fig. 2a). Whilst ambitious afforestation rates of 16 kha/yr from 2030 onwards contribute up to 4.8 Mt CO_2_e of removals to offset agricultural emissions, existing forest becomes a net emission source of circa 2 Mt CO_2_e by 2050 owing to an increase in harvest, a decline in productivity due to age-class profile shifts and continuing GHG emissions from drained histosols (peat soils) on which half of it is planted (Fig. 2a). Meanwhile, “other land use” remains a source of almost 6 Mt CO_2_e owing to a switch from CO_2_ to methane emission following peat soil rewetting^20^. Temporary C storage in wood products and tCDR—proxied as permanent geological storage of CO_2_ following carbon capture from half of all bioenergy by 2050—make decisive contributions to negative emissions, up to 6.3 Mt CO_2_e by 2050 for the split gas BE pathway (Fig. 2c). Continued afforestation and progressive deployment of tCDR through to 2100 results in net emissions of 0.8 (BE) to 7.6 (CN) Mt CO_2_e by 2100 (Fig. 2c).

**Fig. 3:**
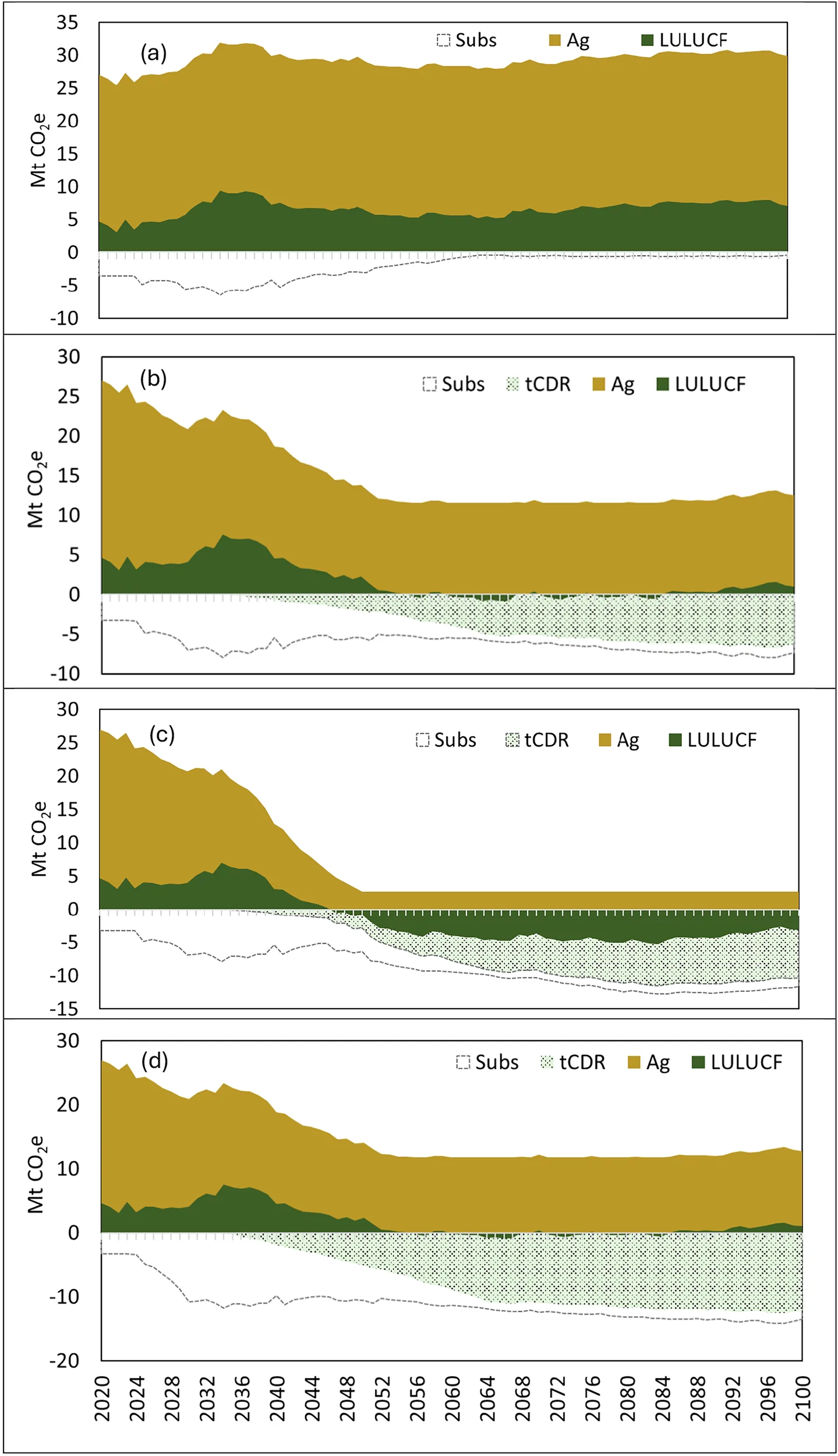
Greenhouse gas emissions and removals time series. Area charts displaying greenhouse gas emission balance through time for business as usual (**a**), Sustainable Intensification achieving split gas climate neutrality by 2050 (**b**), Sustainable Intensification achieving all-gas net zero by 2050 (**c**), and for the Bioeconomy pathway achieving split gas climate neutrality by 2050 (**d**). Harvested wood product sink counted within agriculture, forestry and other land use (AFOLU) emissions balance; technical carbon dioxide removal (tCDR) displayed separately. Downstream substitution effects are shown for context only, and do not count towards AFOLU climate neutrality calculations in back-casting.

Increased soil, biomass and HWP C storage can be counted as “negative emissions” for landuse, landuse change & forestry (LULUCF) under national inventory reporting^20^. Here, we assume that tCDR via bioenergy carbon capture & storage (BECCS) could also count as negative emissions, contributing towards AFOLU climate neutrality. From a life cycle (value chain) perspective, total downstream climate mitigation achieved by wood use in the BE pathway (including indicative substitution of concrete and fossil fuels), would equate to over 11 Mt CO_2_e by 2050 (Fig. 2a). Progressive deployment of tCDR across forestry and wood residues, biomethane and willow results in the split gas BE pathway coming within 1 Mt CO_2_e of all-gas net zero emissions from 2065 onwards (Fig. 3d). This highlights the critical contribution that land use could make towards economy-wide decarbonisation through downstream biomass use, particularly important given that even sustained afforestation at 16 kha/yr from 2030 barely manages to offset the 6 Mt CO_2_e/yr of residual emissions from peat soils, resulting in a negligible LULUCF sink from 2050 onwards (Fig. 3b). Sustained afforestation of 25 kha/yr from 2030 in the net zero SI pathway does result in substantial negative emissions from LULUCF of −2 to −5 Mt CO_2_e from 2051 through to 2100 (Fig. 3c).

### Wider ecosystem effects

**Fig. 4:**
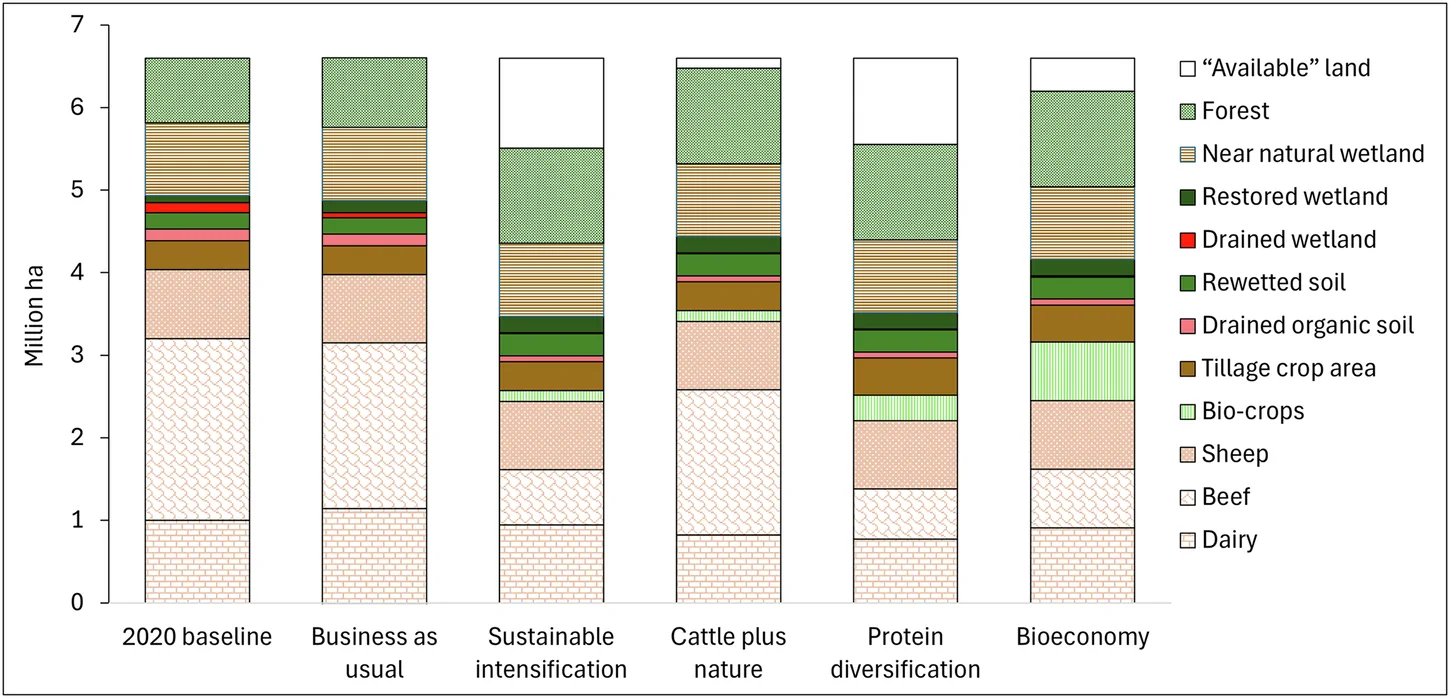
National land areas under major use categories by 2050. Stacked column chart showing areas of major land uses in the 2020 baseline, and for 2050 under business as usual and the four climate-neutral transition pathways (split gas climate target by 2050). Areas are categorised according to 11 primary use or management classes pertinent to greenhouse gas emission accounting, plus an “available land” category that represents land not assigned to a specific type of use or management.

Relative to 2020, total cattle numbers would increase by 6% under BAU, but would decline by between 35% (SI pathway) and 47% (PD pathway) (Table 3). Areas used for beef farming are greatest in the BAU and CN pathways, at 2.0 and 1.8 million ha, respectively, but decline by up to 73%, across other pathways (just 0.6 million ha for beef production in the PD pathway) (Fig. 4 and Table S2). Land areas devoted to dairy and sheep farming remain relatively constant across most pathways, in the region of 1.0 million and 0.8 million ha, respectively, though dairy land area declines by 18% and 22% for CN and PD pathways, respectively. Land under forest increases by almost 50% by 2050 relative to 2020 for all transition pathways. Land areas remaining available for other uses would range from 0 (BAU) up to 1.1 million ha (SI pathway). Under CN, extensive cattle production occupies almost all grassland. Despite producing considerably more food, feed, fibre and energy than the baseline, the BE pathway leaves 0.4 million ha available for non-specified uses (Fig. 4).

**Fig. 5:**
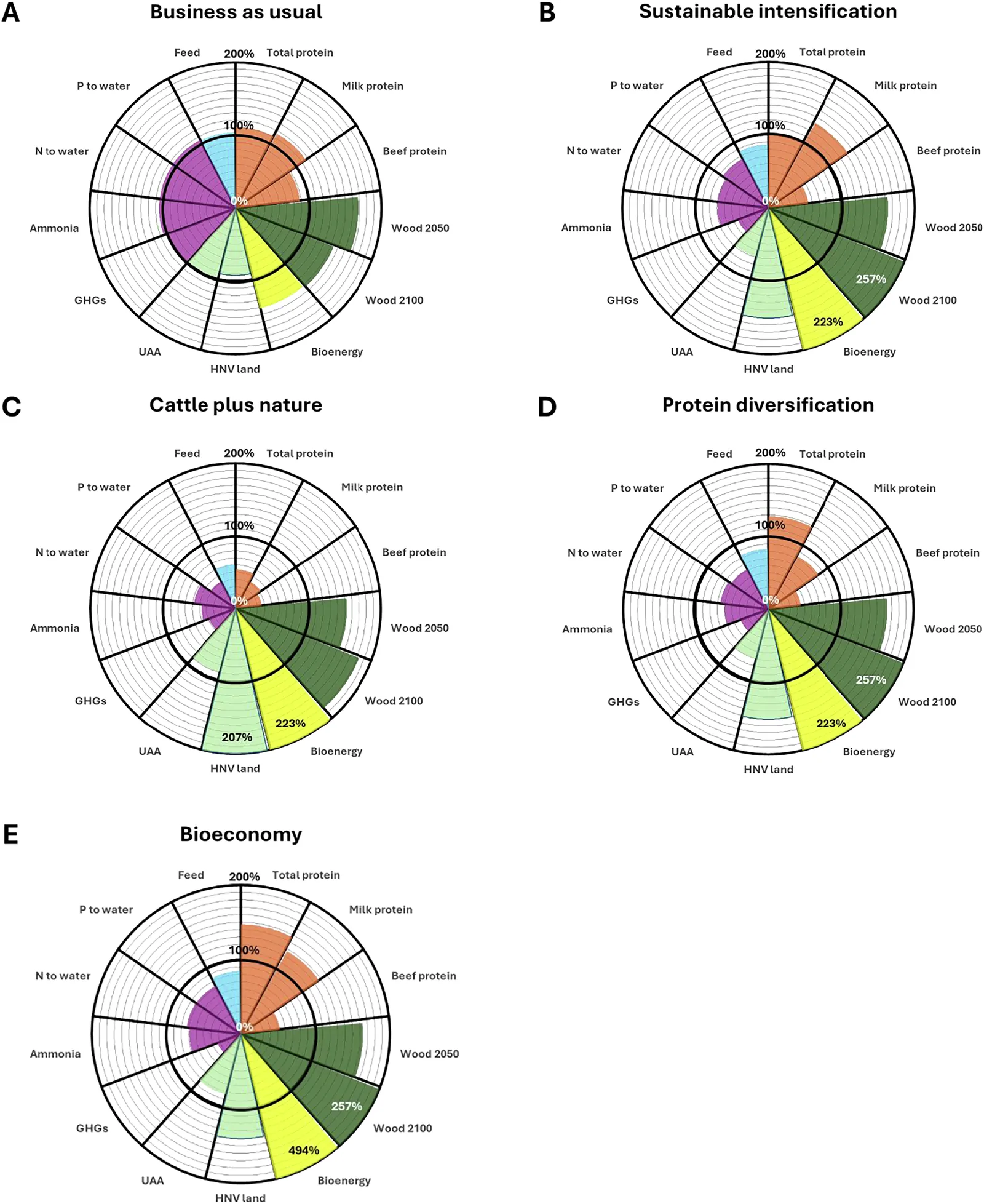
Estimating multifunctionality by 2050. Radar plots displaying multi-dimensional performance of business as usual (**A**) and the four climate-neutral transition pathways (**B**–**E**) under the split gas climate neutrality constraint. 2050 parameter values in Table S2 were normalised against 2020 values as a ratio (100% is represented by inner circle in the figures). Inputs and negative ecosystem services impacts to the left of each panel (values within the inner circle indicate improvement vs 2020); positive outputs (provisioning ecosystem services) to the right of each panel (values outside the inner circle indicate improvement vs 2020). UAA utilised agricultural area, HNV land high nature value land.

Implied ecosystem services effects (positive and negative) largely reflect the aforementioned land balances. Relative to 2020, high nature value (HNV) area would decline by 7% under BAU, but increase by between 41% (BE) and 107% (CN), facilitating compliance with Nature Restoration Law targets. Changes in provisioning services relative to 2020 are presented in Fig. 5. Protein production would be maintained or increased (by up to 50%) across all the pathways that meet split gas climate neutrality, apart from CN. Relative to 2020, milk production would increase by 2050 in the BAU (+ 16%), SI (+ 32%) and BE (+ 27%) pathways (Fig. 5). However, milk production would decrease by 58% in the CN pathway, and by 16% in the PD pathway. Wood production increases by circa 60% across all pathways out to 2050, including BAU—reflecting the age profile of existing forests which is projected to support a large increase in wood harvests over the coming decades^32^. The extent and type of new afforestation will influence wood harvests in 2100, which range from 44% higher than in 2020 for BAU to 157% higher for the SI, PD and BE pathways (Fig. 5). A higher share of broadleaf afforestation in the CN pathway would result in an 82% increase in harvested wood by 2100. Bioenergy output (from forest and wood residues) would increase by 42% between 2020 and 2050 for BAU, by 123% for SI, CN and PD (owing to 5.7 TWh annual biomethane production), and by 394% for the BE pathway owing to 400 kha of willow for bioenergy (for BECCS and/or biochar) in addition to 5.7 TWh biomethane (Fig. 5).

Peat soils would account for almost 6 Mt CO_2_e in 2050 even after ambitious rewetting (“Other land use” in Fig. 2)—owing to high methane emission factors applied to rewetted peat soils in national emissions accounting^20^. Sensitivity analyses (Figure S1) shows that excluding all peat emissions from the AFOLU balance for the SI pathway (as an example) would increase net zero constrained protein production by over 2.5 times, and would increase split gas constrained protein production by 22%. Meanwhile, relative to the split gas target, emissions-constrained protein production would be 27% higher in 2050 when using GWP*, or 14% higher if using a minimum EU effort sharing target equivalent to a 30% reduction in agricultural emissions.

All environmental impacts would increase under BAU, but decrease considerably in each of the climate neutral pathways. Ammonia emissions would reduce by 31% (SI) to 53% (CN), with similar reductions for nitrogen (N) and phosphorus (P) losses to water. The largest estimated reductions in environmental impacts would be for the CN pathway, with the exception of GHG emissions which would decline most in the BE pathway because of negative emissions from biomass-driven tCDR. However, spatial patterns of land use (not considered in the GOBLIN model) will be important for determining localised impacts. Water quality hotspots may remain in intensively farmed regions within the SI, PD and BE pathways.

### Socio-economic consequences

Farming is highly subsidised in Ireland, as across Europe and in other regions, and depends on export-driven demand (trade). Profitability is concentrated in intensive dairy, tillage, pig, and poultry farms^21^. The activities remain at current or higher levels across all indicative pathways apart from CN (Table 1). Meanwhile, economic viability is low for many beef and sheep farms. Suckler-beef activity declines across all non-BAU pathways (Table 1). Socio-economic effects will depend strongly on the wider context in which farming operates, described below under *Resilience*. Taking AFOLU production quantities at face value in Table S2 (they could be impacted by climate change under different SSPs: Table S5), and using recent farmgate prices, BE and BAU output is valued at €16.4 bn and €15.5 bn annually, relative to €14.6 bn for the 2020 baseline under an SSP2 future (Table S4). SI, PD and CN generate lower output values, at €14.5 bn, €12.8 bn and €10.2 bn, respectively (Table S4). Agroecological shifts within the alternative pathways (e.g. extensive use of grass-clover) reduce dependence on imported feed and fertilisers. This could lead to reduced activity across agri-suppliers, as well as beef processors (Table 1). In terms of balance of trade at national and farm sector level, subtracting imported compound feed and fertiliser costs, and GHG compliance costs to offset excess emissions in BAU, all alternative pathways except CN could generate higher net output than BAU for farmers in aggregate (Table S4). Under SSP2, it was assumed all pathways would attract the same circa €2 bn annual farm subsidy paid out in 2024^33^. If future subsidies under SSP1 and SSP4 futures were scaled according to HNV areas, the CN pathway could attract subsidies over €4 bn (Tables S4b and S4d). CN income could also be boosted if private markets in ecosystem service delivery were to develop, which was not estimated^33^. At farm level, SI, PD and BE pathways imply a shift towards land uses that would generate higher family farm income per hectare^21^, and involve more productive animals and land management which would also tend towards improved farm profitability. The high degree of consolidation towards more efficient milk production and shift out of suckler-beef production in the SI pathway implies little or no active farming across tens of thousands of small farms on the 1.1 million ha destocked of animals. In PD and BE pathways, most of the destocked area would be used for alternative farming activities. Realising potential economic benefits at farm-, value chain- and national balance of trade- level would depend strongly on new demand and the development of downstream processing capacity; in turn related to wider decarbonisation and sustainability shifts. These shifts could be associated with new revenue streams and value-added across suppliers of abatement technologies, alternative protein feed and food processors, energy providers and carbon capture and transport companies, as well as companies specialising in peatland rehabilitation and intermediaries for marketable ecosystem service provision (Table 1). Consequences for rural employment would depend on successful development of these value chains, to achieve strong economic multipliers^34^.

**Table 1:** List of the main activities that would significantly expand or contract under the indicative pathways considered, undertaken by existing or new actors

Indicative pathway	Existing actors		New actors
	Activities contracting	Activities expanding	Activities expanding
Business as Usual	Little change	Dairy cow numbers and output; Limited forestry expansion	Few new activities
Sustainable Intensification	Suckler-beef farming; Beef processing; Fertiliser & feed sales	Dairy output—through more productive animals; Large forestry expansion (commercial and conservation); Abatement technology sales Peatland rehabilitation	Anaerobic digestion; Frontier abatement technology sales (e.g. methane inhibitors); Wood residues heat and electricity generation; Some CO_2_ capture and transport and/or biochar production (post 2035)
Cattle + Nature	Dairy farming (fewer animals; Milk and beef processing; Fertiliser & feed sales	Large forestry expansion (commercial and conservation); Peatland rehabilitation	Payment for ecosystem services schemes; Anaerobic digestion; Wood residues heat and electricity generation; Some CO_2_ capture and transport and/or biochar production (post 2035)
Protein Diversification	Suckler-beef farming; Beef processing; Fertiliser & feed sales	Dairy output—through more productive animals; Pig & poultry production; Tillage cropping; Large forestry expansion (commercial and conservation); Abatement technology sales Peatland rehabilitation	Grass biorefining; Alternative protein cropping & processing; Anaerobic digestion; Frontier abatement technology sales (e.g. methane inhibitors); Wood residues heat and electricity generation; Some CO_2_ capture and transport and/or biochar production (post 2035)
Bioenergy	Suckler-beef farming; Beef processing; Fertiliser & feed sales	Dairy output—through more productive animals; Large forestry expansion (commercial and conservation) Tillage cropping Abatement technology sales Peatland rehabilitation	Energy crop cultivation; Grass biorefining; Alternative protein cropping & processing; Anaerobic digestion; Frontier abatement technology sales (e.g. methane inhibitors); Wood residues and energy crop heat and electricity generation; Ambitious CO_2_ capture and transport and/or biochar production (post 2035)

### Resilience and risk

**Fig. 6:**
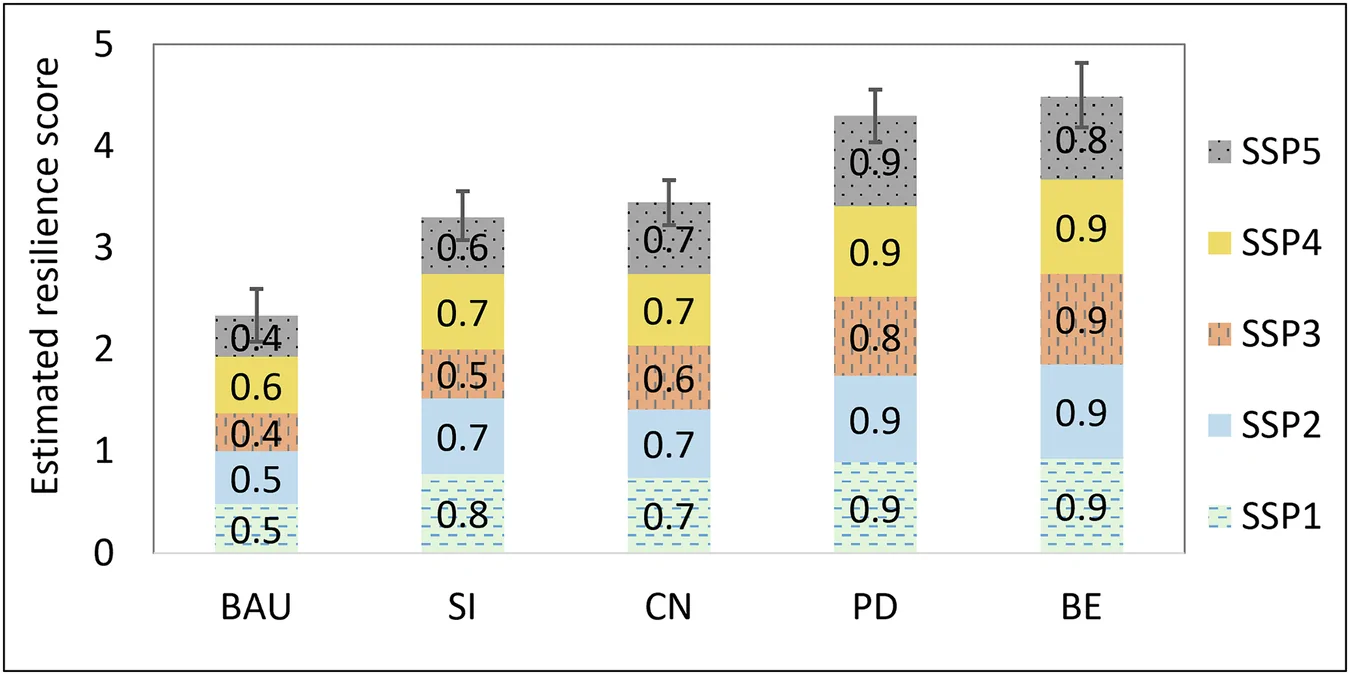
Estimating resilience under different futures. Resilience scores for each indicative pathway under each of the five Shared Socioeconomic Pathway (SSP) future contexts. Scores are derived from structured, qualitative screening involving assignment of a 1–3 high- to low-risk categorisation across the nine risk factors described in Table 5 (full details of underlying scoring in Table S5). Scores are normalised against the maximum possible score to give a maximum resilience score of 1 within each future context, and 5 in aggregate (i.e. higher scores infer higher resilience). Error bars represent 95% confidence intervals in aggregate scores, following Monte Carlo simulation of 25% of assigned scores changing ±1 (within 1–3 bounds), indicating robust ranking apart from some interchangeability between the Sustainable Intensification (SI) and Cattle + Nature (CN) pair, and between the Protein Diversification (PD) and Bioeconomy (BE) pair.

Figure 6 summarises the performance of the five pathways within uncertain future SSP global contexts, based on aggregate low (1) medium (2) or high (3) scores across nine risk factors (Table 5)—scoring elaborated in Table S5. The ranking of pathways is insensitive to three alternative weighting approaches described in Methods, though a mini-max approach switches BE and PD between first and second rankings. 95% confidence intervals derived from Monte Carlo variation of 25% of scores confirm that overall rankings are robust, though there could be limited specific reversals between SI and CN, and between PD and BE. BAU performs poorly across all SSP futures (scoring 0.4 to 0.6 across SSPs). BAU is highly exposed to private and public costs incurred by regulatory risk in a sustainable future (SSP1), trade risk in a regional rivalry future (SSP3), un-competitiveness of small farms in the inequality (SSP4) and climate impacts in the fossil-fuelled development (SSP5) futures (Table S5). Internalisation of external costs via policies such as carbon pricing could cost farmers over €6 bn in SSP1 and SSP4 (Table S4). BAU faces a combination of moderated risk across these factors in a “middle of the road” (SSP2) future (score 0.5). A regional rivalry future (SSP3) incurs the lowest scores across all pathways owing to the large impact of restricted trade on Ireland’s export-driven agri-food sector. BAU and SI pathways are particularly vulnerable (scores of 0.4 and 0.5, respectively; Fig. 6). The BE pathway is the best performing pathway overall (aggregate score 4.5), closely followed by the PD pathway (aggregate score 4.3). These pathways benefit from diversified outputs which mitigate risks and increase resilience. A specific risk hotspot for the BE pathway is failure to commercially scale BECCS, though this could be mitigated by alternative tCDR options such as less-efficient but lower-tech pyrolysis to produce biochar under a future context requiring tCDR (e.g. high carbon pricing). Biophysically resilient owing to low intensity grassland use and ample buffer capacity for shocks, but with low baseline output and a lack of diversity in marketable outputs, CN performs modestly overall (aggregate score 3.4). SI performs similarly (aggregate score 3.3), owing to investment and additional revenue being concentrated across circa 12% of farms that are assumed to intensify and expand in this pathway—concentrating private, public and systemic risk whilst threatening wider rural employment and community viability.

Important information underpinning this risk screening includes private and public investments associated with the pathways (Table S3). Notably, BAU involves considerable private farmer investment of almost €1.5 bn annually in farm infrastructure and machinery^21^, despite low levels of economic viability across most farms, and could incur public (and possibly private) carbon costs of €2.7 bn annually by 2050 under SSP 1 and 2 (Table S4). Farmer investments are often supplemented with government grant-aid. In combination, these factors contribute to medium- to high-risks for private insolvency, public fiscal exposure and public stranded assets under most SSP futures (Table S5). Conversely, the BE transition requires the largest annualised investment (€2.3–€2.9 bn), split 67% to 33% between farmers and public funds (Table S3). However, owing to the contribution of bioenergy to energy security and income diversification, and tCDR to both climate mitigation and food security (by offsetting more agrifood emissions within climate neutrality constraints), this investment supports revenues (Table S4) and reduces aggregate risk for the BE pathway (Fig. 6). The PD pathway requires less investment and lower dependence on tCDR to deliver a similar resilience score to the BE pathway.

## Discussion

Strategic foresight combined with back-casting of climate neutrality targets demonstrates that BAU for Ireland’s AFOLU sector is both unsustainable and risky for farmers and taxpayers alike. Over the past 50 years Ireland’s farming system has trended towards high specialisation in cattle to produce milk and beef. AFOLU modelling indicates that BAU is likely to increase GHG and ammonia emissions, leaving Ireland adrift of regulatory emissions targets and international climate change action commitments—potentially undermining the hitherto successful green marketing of Irish dairy and beef exports that have driven cattle expansion^35^. A trend of declining water quality is likely to persist through increased nutrient loading, whilst HNV area could decline. None of the four alternative, archetypal climate-neutral transition pathways explored in this study is intended to offer a singular “solution” to the multifaceted challenges facing Ireland’s AFOLU sector in a changing world. Rather, multi-dimensional evaluation of these simplified pathways provides new evidence on trade-offs and co-benefits, risks and opportunities, associated with prioritising particular objectives from land. There remains a need to explore a much wider spectrum of possible pathways^36,37^. Nonetheless, some policy-relevant conclusions can be supported from this study:

1. The diversification central to climate-neutral AFOLU transitions in Ireland could deliver wider sustainability and resilience benefits, mitigating possible future risks arising from disruptions in trade, regulations and/or climate. Foresight analysis can elucidate these risks for stakeholders, providing important counterbalancing evidence to the tangible risks of change for many existing actors (Table 1).
2. Food production can be maintained or increased from a sustainable and resilient AFOLU sector, but steering towards the necessarily constrained “solution space” is likely to require strong regulatory and fiscal interventions. For example, afforestation rates of 16,000 ha per year in climate neutral pathways are eight times the current planting rate (despite generous grants) and twice the policy target^38^. Interventionist policies such as public land purchases for planting, and an agricultural GHG tax to disincentivise high-emission land use, merit consideration given multi-billion € annual public finance liabilities associated with climate regulation non-compliance.
3. Coordination of strategic land use (ambitious peatland rewetting, afforestation, bioenergy crop cultivation) and bio-industrial (cascading wood use, tCDR^39^) policies will be critical to offset agri-food sector emissions and energy-import dependencies. Development of a material and energy bioeconomy is not in competition with the agri-food sector, but a critical strategy to reduce common private, public and systemic risks.
4. Agroecology principles enhance ecosystem services delivery and resilience^37^, and could play a role in the sustainable intensification that is core to three of the climate-neutral pathways. However, there could also be trade-offs between sustainable intensification (more food, less resilience) and agroecology (less food, more resilience) paradigms, at scale. For example, broadleaf forests and extensive cattle grazing in the Cattle + Nature pathway would enhance biodiversity and biophysical resilience, but reduce food output and weaken climate mitigation^40^ relative to the conifer-dominated forests and more intensive dairy cattle systems within the other transition pathways. This pathway poses a particular risk of environmental impact leakage via displacement of food production overseas.
5. Ireland is well positioned to exploit market opportunities for alternative food- and feed-proteins with lower environmental footprints than bovine protein, which could alleviate aforementioned trade-offs.
6. A spatially-targeted combination of the actions bundled within indicative archetypal pathways considered here is likely to best deliver multifaceted objectives from land, and will require spatial analyses (within the national “solution space” envelope we have begun to delineate here).

Extrapolating scientific outputs from national AFOLU modelling into a broader risk framework that embraces future uncertainty is the central innovation of this study. Predictable near-term risks from EU climate regulation (non-)compliance are already on the policy radar^41^. There is less evidence of systematic evaluation of more volatile risks from climate impacts on farm productivity, global trade restrictions, and/or demand shifts. Farm economic viability, already volatile^21^, is highly exposed to such risks. Maintaining viability across over 133,000 mostly family farms in Ireland, with an average farmer age of 59 years old, is a policy priority^21^ that could be in tension with systems transformations required to achieve greater sustainability and resilience. Social and cultural dimensions are critical. A strong pivot towards intensive dairy farming with abatement technology could contribute to future food, revenue and climate objectives, but create community cohesion and resilience risks. Conversely, the Cattle + Nature pathway could maintain existing farm structures and community cohesion, but implies private or public payments to farmers for the delivery of ecosystem services^42^, and substantially reduced food production. Meanwhile, the Bioeconomy and Protein Diversification pathways include diversified revenue streams that could support widespread part-time farming in the landscape, filling the gap left by sustainable intensification alone. The Bioeconomy pathway performs particularly well across food, energy and climate mitigation, reducing exposure to multiple risks such as future GHG emission costs^43^. Technical CDR is central to this performance, but is predicated on significant investment that carries its own risk, and will need to be carefully targeted with regulatory oversight in order to: (i) successfully out-scale technology such as BECCS which remains in early stages of commercial deployment^44^, and (ii) deliver genuine negative emissions in a verifiable manner that counts towards carbon budgets. Proper assessment and communication of these risks in the context of coherent future visions is vital to underpin effective and inclusive policy-making for just farming transitions. Crucially, such risk screening may reframe the perceived balance of risk away from important and tangible short-term investment risks (private and public) associated with AFOLU transitions, towards the multiplicity of severe risks (private, public and systemic) facing all AFOLU stakeholders under plausible future contexts.

Limitations of this study include evaluation of a narrow range of indicative AFOLU pathways that were somewhat conservative by design (i.e. aimed to minimise cattle destocking within climate neutrality constraints). Pathways were deliberately static (non-course-correcting) and archetypal to illustrate trade-offs, and generally predicated on a sustainable intensification paradigm (apart from Cattle + Nature). Future studies could explore a wider set of pathways, including more agroecological approaches often neglected in policy visions^36,37^, and consider dynamic feedbacks (possible course corrections) through time. The GOBLIN model used to evaluate emissions and land use incorporates livestock-land interactions in detail, but is not spatially explicit, limiting the strength of conclusions on nutrient losses to water and biodiversity which are based on simplified aggregate indicators. We focus on AFOLU pathways that comply with a national split-gas climate neutrality target rather than net zero GHG emissions; this reflects the non-net-zero target for global methane emissions under climate scenarios for 1.5 °C^31^ and the particular challenge of circa 6 Mt CO_2_e GHG emissions that persist after rewetting organic soils and peat bogs in Ireland according to revised national inventory accounting methodology^20^. Finally, estimating future socio-economic and climate change impacts across different AFOLU pathways is inherently uncertain, influenced by interacting factors including human agency. Results from the strategic foresight approach here provide insight into major risks and opportunities associated with an indicative range of policy and investment decisions, but cannot provide the (arguably false) precision of economic modelling^18^. Undoubtedly we have missed relevant first- and/or second-order effects in our risk screening. We strongly recommend wider application of strategic foresight approaches—coupled with modelling, risk screening and other methods—to improve the evidence base for AFOLU decision making. Further work is urgently needed to understand the specific institutional and policy changes that would be needed to translate the promising elements of the indicative scenarios modelled here into a just transition for farmers.

For the case of Ireland, we show that there are considerable opportunities for farming to simultaneously support food security, biomaterial and bioenergy production, carbon dioxide removal and delivery of broad ecosystem services—within climate neutrality constraints, and whilst conferring resilience to multiple risks in an uncertain future. However, this will require considerable transformation, and implies a need for radical policies pertaining to carbon pricing, strategic land planning (regulatory approach) and pro-active support for downstream industries such as alternative protein processing, biomaterial production and bioenergy generation (preferably with carbon capture). Farming is continuously evolving in response to a combination of market signals, subsidies and regulation, but change is rate limited. Realising aforementioned opportunities implies a need for far-sighted decision making at multiple levels, and buy-in from a wide range of stakeholders. Translating scientific evidence on the need for transformative change into appropriate action will not be easy. Cultural connections to existing practises, education on effective measures and the dynamics of farm succession are just some critical factors^22^. However, in the context of evolving regulatory pressures, a changing climate and volatile geopolitics, there has never been a more important time for farming stakeholders to critically appraise the direction of travel. It is imperative that such appraisal is based on solid evidence regarding ecological constraints, and appropriate horizon scanning to robustly situate socio-economic risks and opportunities of transition pathways against those of business as usual within a meaningful (decadal) timescale for AFOLU planning. This could help to reframe the considerable and tangible investment and political risks associated with AFOLU transformation.

## Methodology

### Defining indicative pathways

**Fig. 7:**
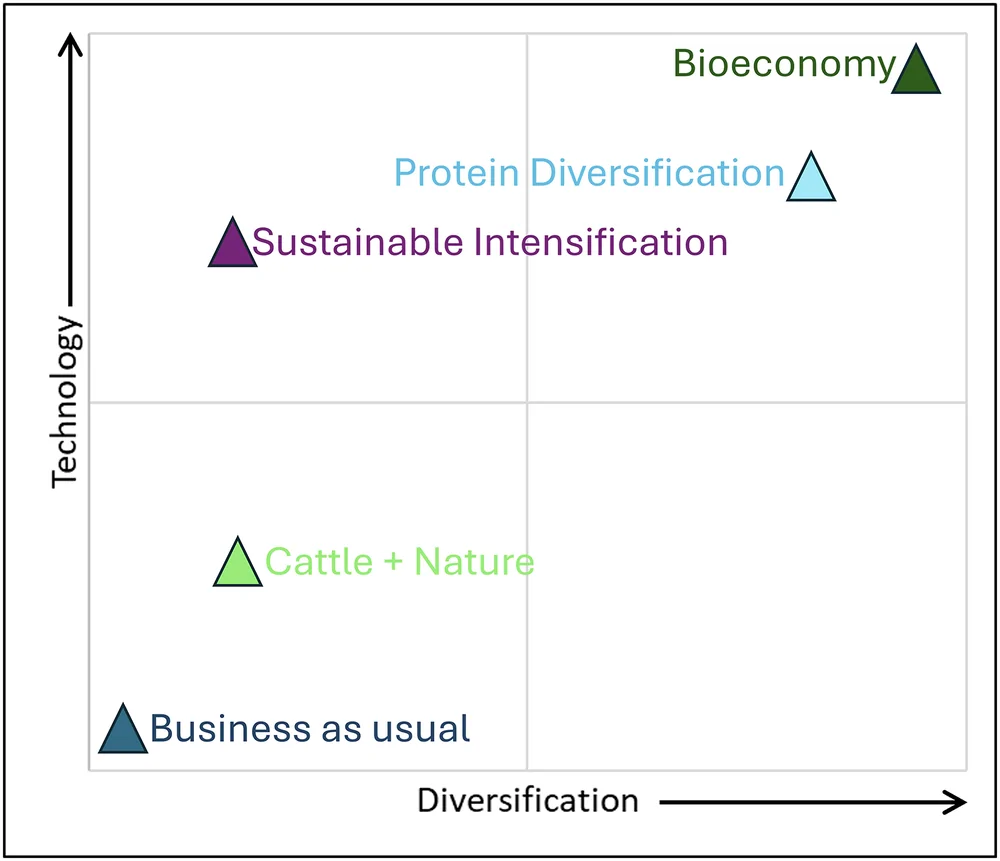
Level of technology deployment and diversification across land use pathways. Scatterplot characterising business as usual and the four climate-neutral agriculture and land use transition pathways according to relative level of diversification (higher towards right) and relative level of technology deployment (higher towards top). The climate-neutral pathways all exhibit more multifunctional land use at landscape scale through additional provision of energy, climate regulation (carbon sequestration and emission offset) and water purification services. Components of these pathways also deliver multifunctionality at field level (e.g. food, biodiversity and water purification from low-intensity grasslands in the Cattle + Nature pathway; energy, climate regulation, water purification and biodiversity from low-input willow cultivation in the Bioeconomy pathway).

Strategic foresight is a systematic means to explore plausible futures, enabling decision-makers to explore how alternative investments and policies pertaining to AFOLU may perform in various anticipated yet uncertain futures^28^. Notably, this approach deliberately moves beyond economic projections to consider how a broad range of possible investment and policy choices could perform in an inherently uncertain future that could look very different from the recent past in terms of trade, markets, environmental (climate) and policy contexts. Accordingly, this study involved the development of four deliberately simplified, archetypal AFOLU pathways that represent different policy choices pertaining to AFOLU. These pathways all embody some degree of multifunctionality at landscape level through the delivery of a wider range of food, fibre, energy, climate regulation, water purification and other ecosystem services. They involve different levels of technology deployment and diversification (Fig. 7) to prioritise: (i) sustainable intensification of protein production from cattle coupled with land sparing—“Sustainable Intensification”; (ii) delivery of ecosystem services through de-intensification of cattle systems—“Cattle + Nature”; (iii) diversification of protein production—“Protein Diversification”, and; (iv) rapid expansion of a bioeconomy that includes substantial bioenergy and technical carbon dioxide removal (CDR)—“Bioeconomy”. Permutations of each of these pathways comply with two definitions of climate neutrality by 2050, and pass through 2030 “waypoints” based on full implementation of Ireland’s Climate Action Plan^38^ (Table S1). These four archetypal pathways are compared with a BAU trajectory that considers minimal progress towards climate, water and biodiversity objectives. This BAU trajectory serves as a low-improvement reference against which the risks and opportunities associated with sustainable and resilient AFOLU transitions can be assessed. It reflects latest emissions projections that show current policies are failing to deliver climate targets^45^—and fall well short of performance needed to operate within planetary boundaries^5^. These AFOLU pathways were then projected into uncertain future contexts proxied to the shared socio-economic pathway (SSP) scenarios used in global integrated assessment models (IAMs)^46^. These SSP scenarios provide a useful framework of alternative future global socio-economic and geopolitical contexts (possibilities), and range from SSP1 (sustainability), through SSP2 (middle of the road) and SSP3 (regional rivalry) to SSP4 (inequality) and SSP5 (fossil-fuelled growth).

Pathways are briefly summarised in the subsequent paragraphs, after explaining their genesis here. The Sustainable Intensification pathway largely reflects AFOLU modelling undertaken to identify agriculture and LULUCF sectoral carbon budgets in Ireland^47^. Three additional pathways were developed through iterative consultation with expert stakeholders in conjunction with back-casting modelling to explore key factors determining compliance with two definitions of Ireland’s legally-binding climate neutrality target^24^ (as one of the most challenging objectives for Ireland’s AFOLU sector). Parameterisation was informed by insight gained from recent randomised modelling of AFOLU net zero pathways^26,27,47^, so that implications of AFOLU choices could be quantitatively explored within (or without) the necessarily constrained envelope of climate neutral futures. A modular approach was taken, involving the distillation of insight from past AFOLU modelling studies into a series of options that could be combined to represent particular priorities (Table 2—elaborated in Table S1). Expert stakeholders included policymakers from Ireland’s Department of Agriculture, Food and the Marine and Department of Climate, Energy and the Environment, scientists from Ireland’s Environmental Protection Agency and Teagasc (agricultural advisory authority), and farming body representatives. Consultation took place over circa five technical working meetings over the period September 2024 to February 2025 and two citizen’s engagement workshops in October 2024 and February 2025 as part of the Land Use Review (Phase 2) process. The development of pathways within an exploratory foresight framework was tentatively linked with possible policy and economic drivers (Table 3) to provide a foundation for private and public risk assessment. Notably, Irish farmers receive circa €2 bn annually in supports, via the European Common Agricultural Policy and Ireland’s exchequer. These include €0.3 bn each from an eco-scheme and a payment for ecosystem services scheme^48^. The remaining €1.4 bn is not tied to environmental measures. In addition, €0.26 bn is available annually to support new afforestation, providing high per-hectare income opportunities but with limited uptake reflecting a variety of socio-cultural barriers to afforestation. Thus, BAU assumes a continuation of just 2 kha annual planting (compared with a peak planting rate of 24 kha in the 1990s) (Table 3). We assume these support payments will extend into the future, with a higher share going towards environmental objectives under SSP1 and SSP2. Additionally, it is assumed that some form of carbon pricing will be needed to drive cattle destocking across pathways (and animal productivity improvements in most pathways), which could be hypothecated to support low-emission technologies and diversification options as in Denmark^49^. Meanwhile, additional government support is assumed necessary to scale out anaerobic digestion and other forms of bioenergy (with carbon capture)—some of the largest capital investment costs in Table S3.

**Table 2:** Steps involved in development of pathways to model within climate target back-casting

Step	Aspect	Options
1	Forest management	Low or high rates of harvest, affecting forest biomass carbon stocks^45^
2	Afforestation rate from 2030 onwards	2, 8, 16 or 25 kha/yr based on BAU, current policy or higher rates previously shown as necessary to achieve different definitions of climate neutrality and within upper bound of historic afforestation rates^47,50^. Continues out until 2080 to bound long-term climate mitigation potential
3	Afforestation species mix	50:50 or 30:70 broadleaf to conifer ratio, representing current policy preference or a commercial conifer preference to provide stronger climate mitigation^40^
4	Peat bog rehabilitation	Fraction of drained area rehabilitated, starting at the 50% recently rehabilitated by Bord na Mona, and moving up to 90% as an assumed technical maximum
5	Rewetting drained organic soils under grass	Fraction of drained organic soil under grass rewetted, from 0% under BAU to the assumed technical maximum of 90% of drained area
6	Anaerobic digestion deployment	Implementation of the ambitious national biomethane strategy to generate 5.7 TWh/yr biomethane (yes or no). Prioritises food waste, animal manures and then grass-clover^47^
7	Pig & poultry activity (production) level	Set at either baseline (2020) production or increased by 20%. NB. Sheep numbers are held at baseline levels across pathways as much of the 0.8 million hectares used for sheep farming is assumed to be upland not well suited to alternative productive uses—a discussed limitation
8	Protein diversification—crops	Additional cultivation of protein crops to produce 150 kt protein, requiring circa 100 kha additional cropping—maximum expansion within good agronomic practise for legume-crop rotation^63^
9	Protein diversification—grass-clover biorefineries	Implementation of grass-clover protein biorefineries to produce 150 kt protein, requiring circa 180 kha grassland^64^
10	Technical carbon dioxide removal	Future deployment of tCDR across biogenic carbon available for energy or biochar conversion—namely biomethane, forestry residues, sawmill residues and end-of-life wood, with progressive deployment at 3% of biogenic carbon sources from 2035 onwards^53^
11	Cattle herd profile	Dairy:suckler cow ratio to determine cattle herd configuration, either 3:2 baseline ratio (in 2020) or 10:1 ratio to prioritise more profitable and land-efficient milk protein production^50^
12	Dairy cow productivity	Medium based on 2020 levels at 14.85 L/day, or High at 19.2 L/day—the 30% increase assumed close to upper bound of animal productivity for grass-dominated diets
13	Beef cow productivity	Medium at Baseline (2020) levels); High assuming a 100-day reduction in average cattle finishing times^50^
14	Technical abatement level	Low at baseline abatement (in 2020); Medium equating to 2030 abatement potential^51^; High equating to “frontier” abatement via full deployment of ambitious technologies such as methane inhibitors and manure emission inhibitors^50^

Decisions made in consultation with stakeholders, informed by previous climate neutrality modelling. Steps 1–10 pre-determine certain AFOLU activities; steps 11–14 parameterise cattle production which is scaled according to emissions budget within specified climate neutrality constraint.

**Table 3:** Key characteristics of the five core AFOLU pathways evaluated in this study, at two critical target years for climate policy (2030 and 2050)

Pathway	2030	2050	Indicative policy interventions
Business as usual	As per 2050, animal number trajectories interpolated linearly.	Afforestation continues at circa 2 kha/yr 50:50 broadleaf:conifer Deforestation continues at 752 ha/yr High rate of forest harvesting No additional rewetting Dairy cow numbers increase to 1.8 million, suckler cow numbers remain similar (0.87 million) based on EPA projections^45^ Medium productivity increase^50^ Emissions intensity held constant Other activities and areas held constant	Existing subsidy supports of circa €2 bn per annum directed towards existing farming systems—includes circa €0.6 bn for agri-environmental measures. Ongoing promotion of abatement measures through agri-advisory support. However, no additional regulation or strong policies such as agricultural emission pricing to drive abatement & diversification. Afforestation grants are financially attractive, but uptake remains low owing to various “lock ins” to current farming and farmer preference to keep options open for future land use.
Sustainable Intensification (split gas climate neutrality target)	Afforestation ramp up to 16 kha/yr 30:70 broadleaf:conifer No deforestation Extended forest harvest interval +65,900 ha peat rewetted^38^ 80,000 ha organic soil under grass rewetted^38^ Full implementation of abatement measures in Teagasc MACC^51^ Biomethane strategy implementation^38^ Cattle numbers scaled to achieve 25% GHG reduction Medium cattle productivity increase^50^	Afforestation 16 kha/yr^47^ No deforestation Extended forest harvest interval 90% rewetting of drained peatland^47^ 90% rewetting organic soil under grass^47^ Frontier ag abatement measures^50^ Biomethane strategy implementation^38^ 2 Mt/yr technical CDR (cascading wood biochar and/or BECCS)^47^ Cattle numbers scaled to achieve split-gas target (10:1 dairy:suckler)^47^ High productivity increase for cattle^47^	Carbon tax or cap & trade to constrain sectoral emissions and leverage diversification (including higher uptake of afforestation grants). Hypothecation of carbon revenue to technological productivity and abatement measures (“sustainable intensification”)^49^, alongside possible herd retirement (exit) scheme. Combined with low gross margins for beef cattle farms, this could drive suckler-beef destocking whilst preserving efficient dairy systems. Land sparing by design—encourages uptake of afforestation grants on spared land. Capex and/or renewable energy and/or bioenergy mandates could drive AD and wood energy deployment.
Cattle + Nature (split gas climate neutrality target)	Afforestation ramp up to 16 kha/yr 50:50 broadleaf:conifer No deforestation Extended forest harvest interval +65,900 ha peat rewetted^38^ 80,000 ha organic soil under grass rewetted^38^ Full implementation of abatement measures in Teagasc MACC^51^ Biomethane strategy implementation^38^ Cattle numbers scaled to achieve 25% GHG reduction Medium cattle productivity increase^50^	Afforestation 16 kha/yr^47^ No deforestation Extended forest harvest interval 90% rewetting of drained peatland^47^ 90% rewetting organic soil under grass^47^ Teagasc MACC abatement^50^ Biomethane strategy implementation^38^ 2 Mt/yr technical CDR (cascading wood biochar and/or BECCS)^47^ Cattle numbers scaled to achieve split-gas target (circa 3:2 dairy:suckler ratio) Medium cattle productivity increase^50^	Carbon tax or cap & trade to constrain sectoral emissions and leverage diversification (including higher uptake of afforestation grants). Hypothecation to payment for ecosystem services (PES), rewarding low intensity grasslands and native broadleaf forestry, alongside a herd retirement (exit) scheme. Cessation of national derogation for organic nitrogen loading under Nitrates Directive, which would limits dairy farm stocking densities. The above policies would favour more extensive (beef) cattle systems and broadleaf afforestation for biodiversity, counterbalancing market drivers for dairy specialisation. Land sharing by design. Capex and/or renewable energy and/or bioenergy mandates could drive AD and wood energy deployment.
Protein Diversification (split gas climate neutrality target)	Afforestation ramp up to 16 kha/yr 30:70 broadleaf:conifer No deforestation Extended forest harvest interval +65,900 ha peat rewetted^38^ 80,000 ha organic soil under grass rewetted^38^ Full implementation of abatement measures in Teagasc MACC^51^ Biomethane strategy implementation^38^ Cattle numbers scaled to achieve 25% GHG reduction Medium cattle productivity increase^50^	Afforestation 16 kha/yr^47^ No deforestation Extended forest harvest interval 90% rewetting of drained peatland^47^ 90% rewetting organic soil under grass^47^ Frontier ag abatement measures^50^ Biomethane strategy implementation^38^ 2 Mt/yr technical CDR (cascading wood biochar and/or BECCS)^47^ Cattle numbers scaled to achieve split-gas target (10:1 dairy:suckler)^47^ High productivity increase for cattle^47^ 20% increase in pig & poultry production Additional 100 kha protein cropping 180 kha grass-protein biorefineries^64^	Carbon tax or cap & trade to constrain sectoral emissions and leverage diversification (including higher uptake of afforestation grants). Hypothecation to technological productivity and abatement measures (“sustainable intensification”)^49^, supports for protein crops and protein biorefineries, and a herd retirement (exit) scheme. Combined with low gross margins for beef cattle farms, this could drive suckler-beef destocking whilst preserving efficient dairy systems alongside expansion of alternative protein farming. Land sparing by design—encourages uptake of afforestation grants and alternative protein production on spared land. Capex and/or renewable energy and/or bioenergy mandates could drive AD and wood energy deployment. Extended to incentivise AD-integrated protein biorefineries^64^. This may need to be accompanied by an ambitious industrial bioeconomy development strategy to drive demand and supply chains.
Bioeconomy (split gas climate neutrality target)	Afforestation ramp up to 16 kha/yr 30:70 broadleaf:conifer No deforestation Extended forest harvest interval +65,900 ha peat rewetted^38^ 80,000 ha organic soil under grass rewetted^38^ Full implementation of abatement measures in Teagasc MACC^51^ Biomethane strategy implementation^38^ Cattle numbers scaled to achieve 25% GHG reduction Medium cattle productivity increase^50^	Afforestation 16 kha/yr^47^ No deforestation Extended forest harvest interval 90% rewetting of drained peatland^47^ 90% rewetting organic soil under grass^47^ Frontier ag abatement measures^50^ Biomethane strategy implementation^38^ 2 Mt/yr technical CDR (cascading wood biochar and/or BECCS)^47^ Cattle numbers scaled to achieve split-gas target (10:1 dairy:suckler)^47^ High productivity increase for cattle^47^ Additional 100 kha protein cropping 180 kha grass-protein biorefineries^64^ 400 kha willow for tCDR	Carbon tax or cap & trade to constrain sectoral emissions and leverage diversification (including higher uptake of afforestation grants). Hypothecation to technological productivity and abatement measures (“sustainable intensification”)^49^, supports for protein and energy crops and a herd retirement (exit) scheme. Combined with low gross margins for beef cattle farms, this could drive suckler-beef destocking whilst preserving efficient dairy systems alongside expansion of protein and energy/carbon farming. Land sparing by design—encourages uptake of afforestation grants and diversification on spared land. Capex and/or renewable energy and/or bioenergy mandates could drive AD and wood energy deployment. Extended to incentivise AD-integrated protein biorefineries^64^ and tCDR. This would need to be accompanied by an ambitious industrial bioeconomy development strategy to drive demand and supply chains.

Explicit policy drivers and responses were not modelled within the foresight framework; however, indicative policy interventions aligned with the pathway changes are listed to provide context for understanding private and public risks.

Business-as-usual (BAU) represents a continuation of current practices alongside a small number of mitigation actions for which specific plans are already in place, as per national projections for emissions trajectories^45^. This pathway involves continued gradual expansion of dairy cow numbers to 1.8 million head and a minor reduction in suckler-beef cow numbers to 0.87 million head, by 2050 (Table 3). Afforestation proceeds at a rate of 2 kha/yr with a 50% broadleaf mix, and forests are harvested at a high rate that exacerbates a deteriorating emission profile associated with aging stands planted on peat soils, as per national projections^45^.

Sustainable Intensification (SI) represents improved cattle productivity and maximum deployment of “frontier” abatement technologies to maintain bovine protein output with circa 35% fewer cattle, and a 10:1 dairy to suckler beef cow preference^50^. This pathway would realise the national target for 5.7 TWh biomethane to be produced by 2030 from food waste, animal manures and grass-clover silage. Ninety percent of exploited peatlands would be rehabilitated, and 50% or 90% of drained organic soils under grass would be rewetted through water table management to reduce CO_2_ emissions (depending on the climate neutrality constraint: Table S1). Afforestation rates of 8 kha (no climate neutrality constraint), 25 kha/yr (absolute net zero constraint) and 16 kha/yr (split gas climate neutrality constraint) are assumed from 2030 onwards, comprising 30 to 50% broadleaf species and the remainder conifers depending on the pathway (Table 3). Forest management would be improved by reducing harvest rates to increase carbon storage in LULUCF^51^.

Cattle + Nature (CN) represents a land sharing, multifunctional land use approach in which extensive cattle systems deliver milk and beef alongside multiple ecosystem services. Relative to 2020, livestock productivity would modestly increase through breeding and currently targeted abatement measures would be implemented, but to a lesser degree than in the SI pathway. The national biomethane strategy would be implemented, peatland rehabilitation, organic soil rewetting and forest management would proceed as per the SI pathway (Table 3). Afforestation would comprise 50% broadleaf species to maximise nature value.

Protein diversification (PD) represents maximum cattle productivity gains and abatement technology deployment are assumed as per SI, but involving fewer cattle. An expansion of pig and poultry production, alongside the establishment of 100 kha of protein crops and 180 kha of grass-clover to produce protein from biorefineries, would diversify protein output (Table 3). Biomethane, peatland rehabilitation, organic soil rewetting, forest management and afforestation would proceed as in the SI pathway.

Bioeconomy (BE) incorporates all elements of the PD pathway except the pig & poultry expansion, alongside 400 kha (equivalent to 10% of farmed grassland area) of willow being established as a low-input bioenergy crop that could deliver higher levels of tCDR alongside multiple ecosystem services (ES) via positive land use change^52^.

In the four alternative pathways, it is assumed that biomass from forestry residues, sawmill residues, end-of-life wood products and willow (BE), and biomethane from anaerobic digestion would support energy sector decarbonisation and future tCDR. Default tCDR modelling assumes slow ramp up of bioenergy with carbon capture and storage (BECCS) from 2035 onwards, so that BECCS would account for 48% of biomass combustion by 2050 and a maximum rate of 90% by 2064^47,53^. However, given that energy substitution credits do not count towards the AFOLU GHG balance, results also proxy for net CDR achieved by alternative technologies such as pyrolysis to produce biochar (lending some robustness to results in case of scaling challenges for BECCS).

Elements of outline pathways were then run through the GOBLIN model^30^, and iteratively scaled to identify variations that complied with two definitions of climate neutrality by 2050. Given that cattle production currently dominates land use, protein production and emissions, dairy and beef production levels were treated as target variables for climate neutral pathways—i.e. cattle numbers were scaled in blocks of 10,000 for each pathway at specified dairy:beef herd composition, animal production level and level of abatement technology (Table 3) to identify maximum cattle numbers within land and GHG constraints (more details below).

### GHG emissions modelling and back-casting

Ireland is committed through legislation to achieve a “*climate resilient, biodiversity rich, environmentally sustainable and climate neutral economy by 2050*”^24^. Ireland’s GHG emissions are also regulated under the EU Climate Law^54^, exposing Ireland to high compliance costs for exceedance of national emission targets. The AFOLU sector accounts for over 40% of national GHG emissions^20^, with a large share of these in the form of short-lived methane. Given the dynamics of methane in the atmosphere, global agricultural emissions do not need to be brought to (net) zero, but rather need to be reduced by circa 32% by 2050, to constrain warming to less than 2 *°*C^31^. As a result, various definitions of “net zero” and “climate neutrality” have been proposed^27^. The most widely used definition of climate neutrality by 2050 is net zero GHG emissions, in which the sum of CO_2_e from all emissions is completely offset by CO_2_ emission sinks (“negative emissions”) on a GWP100 basis. Here, CO_2_e was calculated based on values of 1, 28 and 265 for CO_2_, methane and nitrous oxide, respectively^55^. A second split-gas definition of climate neutrality was also applied, recognising the powerful but short-lived warming attributed to emissions of methane which remain in the atmosphere for approximately 12 years. It has been estimated that global climate stabilisation, within the ambition of the Paris Agreement, requires a 32% reduction in methane emissions from agriculture (assuming ambitious decarbonisation across the rest of the economy)^31^. A simple downscaling of this target to Ireland is used to define a split gas climate neutrality definition. Owing to high methane emissions from LULUCF associated with organic soil rewetting, achieving this target requires a 46–48% reduction in agricultural methane emissions across SG climate neutral pathways.

**Fig. 8:**
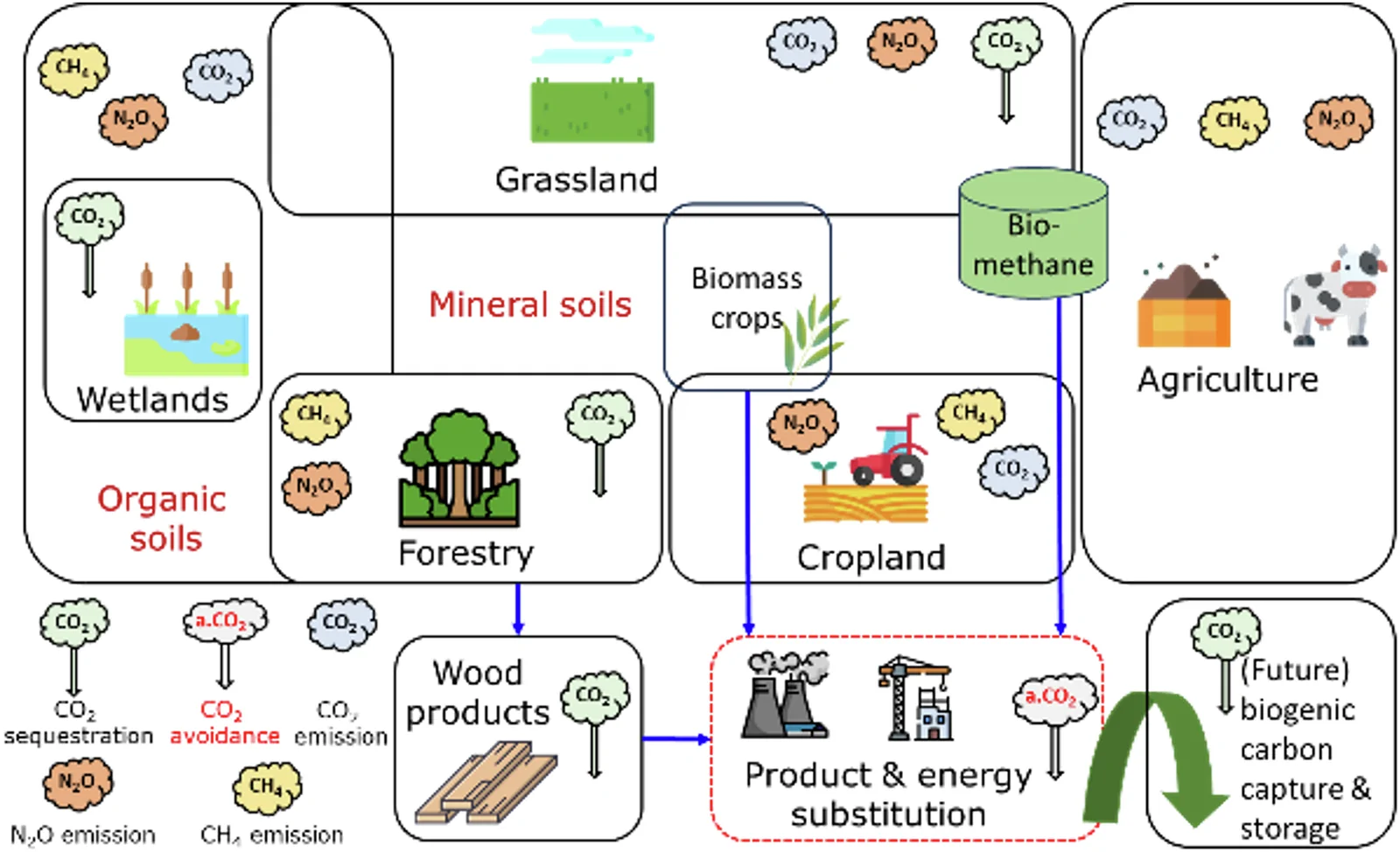
Schematic representation of main sources and sinks of greenhouse gas emissions accounted for in the GOBLIN model. Indicative product and energy substitution values are calculated; these represent downstream greenhouse mitigation and are not counted in the agriculture, forestry and other land use sector.

The biophysical AFOLU back-casting model GOBLIN (Fig. 8) was used to model land and emissions consequences of different pathways^30^, coupled with the life cycle anaerobic digestion (LCAD 2.0) inventory model to include full environmental effects of anaerobic digestion^56,57^. GOBLIN was developed to explore future pathways for Ireland’s AFOLU sector, out to 2100, in terms of production, activity levels and emissions to air and water at national level, and has previously been run for randomised scenarios to identify what net zero could look like for Ireland’s AFOLU sector^26,27,50^. Land classifications and GHG accounting in GOBLIN follow national emission inventory compilation for UNFCCC reporting^20^, incorporating 2019 Refinements to the 2006 IPCC Guidelines for National Greenhouse Gas Inventories^58^. Agricultural emissions are dominated by methane from enteric fermentation and manure management (primarily from cattle), nitrous oxide from manure management and fertilisation of agricultural soils, along with carbon dioxide emissions from the application of lime and urea to soils. The LULUCF sector comprises 7.1 million ha of land, categorised into six managed land categories. Forest land accounts for 773 kha and contributed a removal of ~1.5 Mt CO_2_e in 2022, with an additional removal of ~0.9 Mt CO_2_e from Harvested Wood Products (HWP). Projections indicate that forest land will become a source of emissions during the 2020 s owing to low levels of afforestation since the late 1990s and an increase in the level of harvesting from the private sector, alongside continued emissions from forests on organic soils^20^. Grasslands cover 4.16 million ha and contributed a net 2.5 Mt CO_2_e in 2022, the balance of CO_2_ emissions from drained organic soils under grass minus the CO_2_ sink into mineral soils under grass. Cropland includes all permanent crop and tillage areas (circa 350 kha). It is a minor net sink of GHG emissions (0.08 Mt CO_2_e in 2022). Wetlands represent 1.2 million ha and emitted 3.8 Mt CO_2_e in 2022. Important downstream GHG avoidance arising from agriculture and LULUCF sectors are counted in other sectors, as per the UNFCCC reporting guidelines^58^.

In this study, GOBLIN was run in an iterative, systematic manner. GOBLIN was parameterised to represent three levels of productivity and three levels of emissions abatement for national dairy, beef and sheep systems^50^. The model was then run iteratively to find indicative pathways that achieved two definitions of climate neutrality for the four alternative pathways. Net emissions were first calculated for LULUCF, non-cattle agricultural activities and downstream HWP and tCDR. Then, cattle numbers at given production intensities and abatement levels were iterated in blocks of 10,000 to the maximum allowable within the aforementioned net zero or split gas climate neutrality constraints. To enable this iteration, ratios of dairy cows to suckler-beef cows had to be fixed a priori, along with many other parameters (Table 3). According to the pathway development logic and characteristics explained above, the following sequence of decision steps were taken to parameterise each pathway:

Given some uncertainties over how “climate neutrality” will be defined for Ireland, and some uncertain emission factors for organic soils, we undertook a sensitivity analysis on the SI pathway. A Temperature Neutrality definition of climate neutrality has been proposed for Ireland, based on warming dynamics of changing methane emissions, though is predicated on contentious value judgements that result in reducing emissions of methane being regarded as a national contribution to cooling, even when starting from a high baseline^25^. As a proxy for Temperature Neutrality, we explored what a GWP* target could mean for the SI pathway. Meanwhile, net zero emissions pathways at EU level involve a circa 30% reduction in agricultural emissions by 2050^10^. To provide a contrast with the national net zero target, a speculative “generous” interpretation of this target was applied—based on Ireland matching EU-level percentage changes in sectoral emissions by 2050 (relative to 2015), as an absolute lower bound of effort-sharing by Ireland (see Figure S1).

### Wider environmental and ecosystem services impacts

GOBLIN is not a spatially explicit model. However, it does include activity-specific emission factors for ammonia emissions and mass-balance estimates of nitrogen and phosphorus losses to water^59^. These emissions provide indications of water quality pressure in aggregate at national scale, though require further interpretation of where land use changes are likely to occur to make any inferences about regional water quality pressures. Biodiversity is notoriously difficult to benchmark. Here, we use estimates of high nature value (HNV) land as a proxy for biodiversity (and wider ecosystem services), assigning fractions of particular land uses to *potential* HNV land, namely: all areas of rehabilitated peatlands and rewetted organic soils under grassland, all areas of broadleaf forestry, one third of land under cattle and sheep grazing^60^, all willow area and all “available land” in pathways. In the SI pathway, intensive land use means that none of the diminished beef area is counted as HNV, whilst in the CN pathway all beef area is counted as HNV owing to low stocking densities. Biodiversity impacts of future land use will depend on specific practices. Indicator values are summarised in Table S2.

### Socio-economic outcomes and interaction with Shared Socioeconomic Pathways

Strategic foresight deliberately explores future pathways outside typical bounds of economic models that tend to be constrained by reversion to the mean, and thus BAU bias^18^. As such, this study did not employ an economic model. Instead, a basic screening of economic values of investments, main inputs and main outputs was undertaken to understand the potential economic potential of the alternative pathways relative to BAU. Investment costs may be borne by different entities depending on the policy and market framework. Based on the indicative policy interventions described in Table 3, and traditionally high government transfers to the farming sector, investment costs associated with relevant scales of technology deployment or management practises in each pathway were assigned to either: (i) private farmers or (ii) government (Table S3). This is a simplification, as for example AD investment costs could also be partly borne by private (energy) investors, but it does provide a reasonable and important distinction between farmer and non-farmer liabilities. Information from Table 3 and Tables S2 & S3, along with data extracted from relevant statistical sources, informed estimates of revenues and costs associated with major outputs and (imported) inputs, respectively, in Table S4. These were used to inform risks, including those attributable to trade disruption, in Table S5. Notably, in terms of trade exposure, over 90% of milk and beef is exported. 66% of Ireland’s food and drink exports go to non-EU countries—though 38% end up in the UK and Northern Ireland^61^. Thus, Ireland’s exports are sensitive to future trade disruptions, though somewhat protected by long-standing EU and UK trade agreements/relationships. Table S4a represents main costs and revenues under an SSP2 future. To indicate possible changes associated with SSP futures, relevant costs and revenues were scaled up or down and new costs incurred (e.g. costs associated with air- and water-pollution were internalised to the farming sector in SSP1 and SSP4 futures) (Tables S4b and S4d).

**Table 4:** Shared socio-economic pathway (SSP) narratives and broad implications for Ireland’s AFOLU sector in 2050 (interactions between pathways and SSP contexts elaborated in Table S5)

SSP	Global narrative (six salient dimensions)	Implications for Ireland’s AFOLU sector
1 Sustain-ability	Farming: Improvements in ag productivity; rapid diffusion of best practices Land use: Strong regulations to avoid environmental trade-offs Diet: Low-meat diets Trade: Moderate Energy: Efficiency & renewable focus Climate impact: Low	Reduced demand for meat and high demand for alternative proteins. High demand for alternative energy sources and negative emissions, including BECCS. High reputational and economic costs for emissions and nutrient leakage. High payments for ecosystem services delivery.
2 Middle of the Road	Farming: Medium pace of tech change in ag sector; entry barriers to ag markets reduced slowly Land use: Medium regulations lead to slow decline in the rate of deforestation Diet: Medium meat consumption Trade: Moderate Energy: Fossil fuels with some renewables Climate impact: Moderate	Continued demand for meat and dairy exports, but extra costs of production via climate disruption. Some demand for alternative energy sources including BECCS. Some direct emission costs and some incentive for ecosystem services delivery.
3 Regional Rivalry	Farming: Low technology development, restricted trade Land use: Hardly any regulation; continued deforestation due to competition over land and rapid expansion of agriculture Diet: Medium meat consumption Trade: Limited Energy: Domestic energy focus Climate impact: Moderate	Restricted exports of milk & meat products, but high demand for more diverse indigenous food production. Demand for alternative indigenous energy sources, including bioenergy. Low direct emission costs and little incentive for ecosystem services delivery.
4 Inequality	Farming: Ag productivity high for large scale industrial farming, low for small-scale farming Land use: Highly regulated in mid- and high-income countries, largely unmanaged in low-income countries leading to tropical deforestation Diet: Medium meat consumption (highly variable by income) Trade: Moderate Energy: Diversified Climate impact: Moderate	Continued demand for meat and dairy exports, but extra costs of production via climate disruption. Some demand for alternative protein and energy sources including BECCS. Low direct emission costs but high incentives for local ecosystem services delivery.
5 Fossil-Fuelled Development	Farming: Highly managed, resource-intensive; rapid increase in productivity Land use: Medium regulations lead to slow decline in the rate of deforestation Diet: High meat consumption Trade: High (regional specialisation) Energy: Fossil fuel dominated Climate impact: High	Continued demand for meat and dairy exports, but reduced feasibility of grazing-based production owing to extreme climate disruption and challenging competition with high-tech indoor livestock systems. Limited demand for alternative proteins or alternative energy sources. Low direct emission costs and little incentive for ecosystem services delivery.

### Risk screening

In reality, output volumes and prices are likely to experience large and non-linear swings based on climate impacts, regulations, geopolitics (especially trade agreements or constraints) and myriad market factors. SSP narratives from O’Neill et al.^46^ were distilled into salient dimensions for Ireland’s AFOLU sector (Table 4 and Table S5). Thus, whilst an attempt was made to reflect possible cost and revenue variations for AFOLU sectors in relation to individual SSP futures in Table S4, future risks are more robustly identified using a qualitative approach, in line with “story-telling” methods used to elucidate policy-relevant insight from widely varying SSP futures^62^. In order to evaluate risks under plausible future contexts, BAU and the four alternative pathways with a split gas climate neutrality constraint were subject to a semi-quantitative risk screening linked with a qualitative story-telling approach^46^.

Each pathway was evaluated according to a risk framework comprising nine factors pertaining to three scales of risk (Table 5). Based on biophysical indicators in Fig. 5 and Table S2, estimates of investment costs (Table S3), revenues, costs, import-export balances, carbon costs and externalities (Table S4) and SSP narratives (Table 4), a low-, medium- or high-level of risk was attributed to each of the risks identified in Table 5, for each AFOLU pathway under each SSP context. Table S5 elaborates this approach with key data points and logic used to assign risk levels. Risks were then assigned scores of 1 (high-risk) to 3 (low-risk). Scores were then aggregated under different assumptions: (i) equal weighting for all risks; (ii) double-weighting for private farmer risk; (ii) double-weighting for public risk; (iii) double-weighting for systemic risk. An alternative mini-max approach was also applied to identify AFOLU pathways with fewest high-risk scores (Table S5). Finally, although following structured logic, the screening approach ultimately involves value judgement. To reflect the associated uncertainly, a Monte Carlo analysis was undertaken in which 25% of scores were replaced with a randomly assigned score one higher or lower (but constrained within the 1 or 3 range). Running 50,000 Monte Carlo simulations allowed 95% confidence ranges to be estimated for pathway aggregate scores (Fig. 6).

**Table 5:** Nine types of risk qualitatively screened at high level for each AFOLU pathway in each SSP context (Table S5)

Risk type	Description	
Private farmer risk	Commodity price volatility	Milk, beef, pork, poultry and crop output prices can fluctuate substantially across years. Input costs can also fluctuate. Indicative values shown in Table S4 for aggregate national costs and sub-sector/national revenues. Distributional economic effects across sub-sectors and Ireland’s 133,000 heterogenous farms were not modelled, but sectoral effects were considered qualitatively (Table 1 and Table S5).
	Insolvency	More persistent stress from the above risk, and/or structural shifts towards higher input and/or lower output prices, and/or additional costs such as carbon pricing, and/or mal-investment and stranded assets (bad debt), could lead to farms becoming insolvent. BAU involves annual farmer investment of circa €1.5 bn in capital equipment^21^, almost half of which is made by dairy farmers. Transition pathways also carry private risks in the form of establishment costs and transition cash-flow gaps during transition: willow takes 3–4 years to first commercial harvest; grass-clover biorefinery supply chains require critical mass that individual farms cannot achieve alone; and frontier abatement technologies (e.g. enteric methane inhibitor boluses represent recurring costs with no direct revenue offset at farm level. Indicative investment costs shown in Table S3^21^. Insolvency risk depends on interacting factors specific to heterogenous farm conditions; high-level screening can only estimate lower or higher risk tendencies.
	Climate change impacts	Unabated climate change is likely to exacerbate commodity price volatility, and could permanently shift agro-ecological zones (and thus suit entirely different farming systems)^9^. These impacts are uncertain, and likely to be context (e.g. soil type) specific, thus manifesting differently across individual farms—factors that could not be fully reflected in high level screening.
Public risk	Fiscal exposure	This risk manifests via government budgets, regulatory liability, and public investment decisions. Sustained subsidies to farm systems that are economically unviable without support present risks, though scope to reallocate these subsidies may be somewhat constrained by EU Common Agricultural Policy rules. Compliance cost liability arises from Ireland’s exposure to EU Effort Sharing Regulation targets and future climate regulations. Exceedance of national targets incurs mandatory carbon credit purchases on international markets (Table S4). On the other hand, large capex subsidies investment in biorefineries and
	Stranded investment	Stranded public investment risk arises if public capital is directed towards infrastructure or advisory capacity that pathway transitions make redundant—for example, investment in processing infrastructure configured exclusively for bovine products. Conversely, transition pathways require new public investment in processing capacity, supply chain development, and advisory services that does not yet exist (Table S3). Large capex subsidies may be required for biorefineries and tCDR in the Protein Diversification and Bioeconomy pathways, incurring risks if markets fail to scale.
Systemic risk	Food security	Systemic risks affect the broader economy and society beyond individual farm businesses or public budgets. Food security relates to (national) availability of reasonably priced food, and depends on indigenous production, imports from open markets and exports via open markets to support international food security.
	Balance of trade exposure	Trade exposure is particularly acute for Ireland’s agrifood sector given that 90% of milk and beef production is exported, creating a high degree of dependency on open markets and stable international demand. Conversely, Ireland imports all fertilisers and a large share of livestock feed. The value of some of these trades can be inferred from Table S4.
	Rural employment & community viability	Rural employment and community viability are central political-economy constraints on all transition pathways. Consolidation under SI implies little or no active farming across the 1.1 million ha destocked of animals, with significant consequences for rural employment and community structures in affected regions. The PD and BE pathways maintain more distributed land use and offer diversified revenue streams that could support widespread part-time farming, but this depends on successful development of new value chains whose employment multipliers are not yet established (Table S4). Availability of appropriately-skilled labour is not explicitly addressed and could be a limiting factor in practice without adequate retraining schemes.
	Environmental externality risk	Environmental externality risks are the social costs of pollution and ecosystem degradation imposed on society that are not internalised in market prices or farm-level accounts. These include water quality deterioration from nitrogen and phosphorus loading, air quality impacts from ammonia emissions, and biodiversity loss from land use intensification and habitat fragmentation. Some indicative external costs are shown in Table S4.

Screening considers aggregate level risk tendencies for different categories of actor at national scale.

## Supplementary information


Supplementary Information_Styles_10.06.2026
Supplementary_Data_09.04.2026


## Data Availability

Model and risk screening results from this study are available in Supplementary Data and Supplementary Information files. Pathway-specific GOBLIN model parameterisation is displayed in Supplementary Information Table S1; full GHG emission time series 2020–2100 are available in Supplementary Data Tables S1–S5; sensitivity analyses around different climate neutrality targets and accounting approaches are displayed in Supplementray Information Figure S1; wider key performance indicator data are summarised in Supplementary Information Table S2; investment cost estimates are summarised in Supplementary Information Table S3; indicative economic screening of pathways under different SSPs displayed in Supplementary Data Table S4 and risk screening summarised in Supplementary Data Table S5.
