# Supplementary material for: Strategic foresight provides insight into the resilience of climate-neutral farming transitions in a volatile world
Source: NPJ Sustain Agric. 2026 Aug 31;4(1):73. doi: 10.1038/s44264-026-00185-2 (PMC13529586; doi:10.1038/s44264-026-00185-2)
Supplement: Supplementary file 1 [file 44264_2026_185_MOESM1_ESM.docx]

Supplementary Information for:

Strategic foresight provides insight into the resilience of climate-neutral farming transitions in a volatile world

David Styles^1^, Daniel Henn^1^, Colm Duffy^1^, Kevin Black^2^, Andres Martinez-Arce^1^

^1^School of Biological & Chemical Sciences and Ryan Institute, University of Galway, Galway Ireland, H91 TK33

^2^Forest, Environmental Research & Services (FERS) Ltd, Sillogue, Kilberry , Navan, Ireland

Corresponding author: David Styles. Email: David.styles@universityofgalway.ie

**Table S1.** Summary of key parameters selected in consultation with expert stakeholders (or deduced via back-casting) for business as usual and the four alternative transition pathways, unconstrained or constrained by one of two definitions of climate neutrality - net zero CO_2_e, or NZ for long-lived gases with a with a separate split gas target for methane.

**Table S2.** Summary of key performance indicators for Ireland's AFOLU sector in 2020, and in 2050 for business as usual (BAU) and the four alternative transition pathways (Sustainable Intensification - SI, Cattle + Nature - CN, Protein Diversification - PD, and Bioeconomy - BE) under two definitions of climate neutrality (net zero CO_2_e “NZ) or net zero for long-lived gases with a with a separate split gas (SG) target for methane. Results from GOBLIN model after parameterisation as per Table S1.

|  | **Areas** | | | | | | | | | | | | | **Environmental impacts** | | | **Outputs** | | | | | | **Input** |
| --- | --- | --- | --- | --- | --- | --- | --- | --- | --- | --- | --- | --- | --- | --- | --- | --- | --- | --- | --- | --- | --- | --- | --- |
| **Year or pathway (2050)** | **Dairy area** | **Beef area** | **Sheep area** | **Biogas grass-clover** | **Biorefinery grass-clover** | **Willow** | **Tillage crop area** | **Drained organic soil** | **Rewetted organic soil1** | **Drained wetland area** | **Restored/rehabilitated wetland area** | **Forest area** | **Available land** | **Ammonia** | **N to water** | **P to water** | **Protein total** | **Wood harvest million m3** | **Total Bioenergy** | **AD bioenergy** | **Forest bioenergy** | **Willow bioenergy** | **Concentrate feed** |
|  | 1000 ha | | | | | | | | | | | | | kt | | | | M m3 | TWh energy | | | | kt |
| **2020** | 999 | 2,203 | 834 | 0 | 0 | 0 | 350 | 141 | 198 | 125 | 85 | 781 | 0 | 106 | 142 | 5.7 | 627 | 3.9 | 6.5 | 0.0 | 9.2 | 0.0 | 4,379 |
| **BAU** | **1,141** | **2,008** | **828** | **130** | **0** | **0** | **350** | **141** | **198** | **62** | **148** | **841** | **-131** | **111** | **149** | **6.0** | **654** | **6.6** | **9.2** | **0.0** | **9.2** | **0.0** | **4,541** |
| **SI** | 1,359 | 1,599 | 828 | 130 | 0 | 0 | 350 | 71 | 268 | 12 | 198 | 989 | -89 | 127 | 153 | 7.6 | 900 | 5.8 | **14.4** | 5.7 | 8.7 | 0.0 | 5,202 |
| **SI_NZ** | 77 | 54 | 828 | 130 | 0 | 0 | 350 | 15 | 324 | 12 | 198 | 1,361 | 2,367 | 12 | 37 | 0.4 | 186 | 6.2 | **14.7** | 5.7 | 9.0 | 0.0 | 1,654 |
| **SI_SG** | **946** | **669** | **828** | **130** | **0** | **0** | **350** | **71** | **268** | **12** | **198** | **1,155** | **1,089** | **73** | **99** | **4.3** | **640** | **6.4** | **14.5** | **5.7** | **8.8** | **0.0** | **3,788** |
| **CN** | 826 | 1,756 | 828 | 130 | 0 | 0 | 350 | 15 | 324 | 12 | 198 | 989 | 288 | 50 | 83 | 2.4 | 337 | 5.8 | **14.4** | 5.7 | 8.7 | 0.0 | 2,644 |
| **CN_NZ** | 0 | 0 | 214 | 130 | 0 | 0 | 350 | 15 | 324 | 12 | 198 | 1,361 | 3,112 | 4 | 19 | 0.0 | 136 | 6.0 | **14.6** | 5.7 | 8.9 | 0.0 | 1,465 |
| **CN_SG** | **826** | **1,756** | **828** | **130** | **0** | **0** | **350** | **71** | **268** | **12** | **198** | **1,155** | **122** | **50** | **83** | **2.4** | **337** | **6.0** | **14.5** | **5.7** | **8.8** | **0.0** | **2,644** |
| **PD** | 1,359 | 1,062 | 828 | 130 | 180 | 0 | 450 | 15 | 324 | 12 | 198 | 989 | 168 | 105 | 137 | 6.2 | 1053 | 5.8 | **14.4** | 5.7 | 8.7 | 0.0 | 5,097 |
| **PD_NZ** | 30 | 23 | 828 | 130 | 180 | 0 | 450 | 15 | 324 | 12 | 198 | 1,361 | 2,165 | 10 | 39 | 0.1 | 485 | 6.2 | **14.7** | 5.7 | 9.0 | 0.0 | 1,831 |
| **PD_SG** | **774** | **605** | **828** | **130** | **180** | **0** | **450** | **71** | **268** | **12** | **198** | **1,155** | **1,045** | **63** | **94** | **3.6** | **803** | **6.4** | **14.5** | **5.7** | **8.8** | **0.0** | **3,660** |
| **BE** | 1,032 | 925 | 828 | 130 | 180 | 400 | 450 | 15 | 324 | 12 | 198 | 989 | 233 | 76 | 110 | 4.2 | 863 | 5.8 | **32.0** | 5.7 | 8.7 | 17.6 | 3,861 |
| **BE_NZ** | 272 | 212 | 828 | 130 | 180 | 400 | 450 | 15 | 324 | 12 | 198 | 1,361 | 1,334 | 28 | 57 | 1.2 | 588 | 6.2 | **32.3** | 5.7 | 9.0 | 17.6 | 2,133 |
| **BE_SG** | **910** | **711** | **828** | **130** | **180** | **400** | **450** | **71** | **268** | **12** | **198** | **1,155** | **403** | **73** | **104** | **4.2** | **921** | **6.4** | **32.1** | **5.7** | **8.8** | **17.6** | **3,701** |

**Table S3.** Indicative investment costs associated with business as usual and the four climate-neutral transition pathways (based on split gas climate target). Investments scaled to relevant activity levels, annualised where necessary based on 20-year loan repayments at 5% interest (blue numbers).

| **Investment item** | **Unit cost (€)** | **Who pays?** | **Business as Usual** | | **Sustainable Intensification** | | **Cattle + Nature** | | **Protein Diversification** | | **Bioeconomy** | | **Notes & key references** |
| --- | --- | --- | --- | --- | --- | --- | --- | --- | --- | --- | --- | --- | --- |
| **Existing ongoing investment – whole sector** | | | | | | | | | | | | |  |
|  |  |  | **Low** | **High** | **Low** | **High** | **Low** | **High** | **Low** | **High** | **Low** | **High** |  |
| **Ongoing investment (annualised)** | €1.49 bn / yr | Farmers | **€1.49** | | **€1.49** | | **€1.49** | | **€1.49** | | **€1.49** | | Conservatively assume same ongoing investment across farms in transition pathways, even though cattle farming contracts in all transition pathways. eagasc (2025). <https://teagasc.ie/wp-content/uploads/uploads/media/website/publications/2025/NFSReport2024.pdf> |
| **Afforestation establishment** | | | | | | | | | | | | | |
| **Establishment grants** | €3,858–6,744 / ha | Public | 50 kha | | 373 kha | | 373 kha | | 373 kha | | 373 kha | | DAFM (2025): <https://www.gov.ie/en/department-of-agriculture-food-and-the-marine/publications/afforestation-scheme/> |
| Total cost | bn € |  | **€0.19** | **€0.34** | **€1.44** | **€2.52** | **€1.44** | **€2.52** | **€1.44** | **€2.52** | **€1.44** | **€2.52** |  |
| Amortised (20 yrs, 5% interest) | **€ bn/yr** |  | **€0.015** | **€0.027** | **€0.115** | **€0.202** | **€0.115** | **€0.202** | **€0.115** | **€0.202** | **€0.115** | **€0.202** |  |
| **Anaerobic digestion & biomethane (5.7 TWh target)** | | | | | | | | | | | | | |
| **Total AD sector capital (5.7 TWh)** | €1.1-1.7 bn total | Public | 0 | | 5.7 TWh | | 5.7 TWh | | 5.7 TWh | | 5.7 TWh | |  |
| Total cost | bn € |  | **NA** | | **€1.13** | **€1.65** | **€1.13** | **€1.65** | **€1.13** | **€1.65** | **€1.13** | **€1.65** | Sahota et al. (2026) <https://www.sciencedirect.com/science/article/pii/S0196890426002852> |
| Amortised (20 yrs, 5% interest) | **€ bn/yr** |  | **NA** | | **€0.091** | **€0.132** | **€0.091** | **€0.132** | **€0.091** | **€0.132** | **€0.091** | **€0.132** |  |
| **Short rotation coppice willow (Bioeconomy pathway only)** | | | | | | | | | | | | | |
|  |  |  | **0 kha** | | **0 kha** | | **0 kha** | | **0 kha** | | **400 kha** | |  |
| **Willow SRC establishment (site prep, cuttings, planting, year-1 weed control)** | €2000–3000/ha | Farmer | **NA** | | **€0.00** | | **€0.00** | | **€0.00** | | **€0.80** | **€1.20** | Teagasc: <https://teagasc.ie/wp-content/uploads/media/website/publications/2010/859_Willow.pdf> |
| Amortised (20 yrs, 5% interest) | **€ bn/yr** |  | **NA** | | **€0.000** | | **€0.000** | | **€0.000** | | **€0.064** | **€0.096** |  |
| **Peatland rehabilitation and organic soil rewetting** | | | | | | | | | | | | | |
| **Peat bog rehabilitation / rewetting (drain blocking, re-profiling) initial cost** | €1,450–3,273/ha | Public | 0 | | 138 kha | | 138 kha | | 138 kha | | 138 kha | | Teagasc (2023): <https://teagasc.ie/environment/climate-centre/publications/reports/marginal-abatement-cost-curve-2023/> |
|  |  |  | **NA** | | **€0.20** | **€0.45** | **€0.20** | **€0.45** | **€0.20** | **€0.45** | **€0.20** | **€0.45** |  |
| Amortised (20 yrs, 5% interest) | **€ bn/yr** |  | **NA** | | **€0.016** | **€0.036** | **€0.016** | **€0.036** | **€0.016** | **€0.036** | **€0.016** | **€0.036** |  |
| **Grassland organic soil rewetting - initial cost** | €400-1000/ha | Public | 0 | | 127 kha | | 127 kha | | 127 kha | | 127 kha | | Teagasc (2023): <https://teagasc.ie/environment/climate-centre/publications/reports/marginal-abatement-cost-curve-2023/> |
|  |  |  | **NA** | | **€0.05** | **€0.10** | **€0.05** | **€0.10** | **€0.05** | **€0.10** | **€0.05** | **€0.10** |  |
| Amortised (20 yrs, 5% interest) | **€ bn/yr** |  | **NA** | | **€0.004** | **€0.008** | **€0.004** | **€0.008** | **€0.004** | **€0.008** | **€0.004** | **€0.008** |  |
| **Frontier abatement technologies (SI, PD, BE pathways)** | | | | | | | | | | | | | |
| **Sum of technical measures (whole sector, annual)** | €58-116 / LU (adult cows) | Farmer | 0 | | 1728 k LU | | 996 k LU* | | 1415 k LU | | 1663 k LU | | Teagasc (2023): <https://teagasc.ie/environment/climate-centre/publications/reports/marginal-abatement-cost-curve-2023/> and Ofori et al. (2026) Marginal Abatamenet Cost Curve for Ireland's AFOLU sector (report to DCEE) |
|  | *€29-€58 / LU for C+N pathway (lower adoption) |  | NA | | **€0.10** | **€0.20** | **€0.03** | **€0.06** | **€0.08** | **€0.16** | **€0.10** | **€0.19** |  |
| **Protein crop & grass-clover biorefinery infrastructure (PD and BE pathways)** | | | | | | | | | | | | | |
| **Grass-clover biorefineries capital investment** | €1.1-1.7 bn total | Farmer | 0 | | 150 kt protein | | 150 kt protein | | 150 kt protein | | 150 kt protein | | Similar dry matter processing volume to AD plants in biomethane strategy - proxy as same investment |
|  |  |  | **NA** | | **€1.13** | **€1.65** | **€1.13** | **€1.65** | **€1.13** | **€1.65** | **€1.13** | **€1.65** |  |
| Amortised (20 yrs, 5% interest) | **€ bn/yr** |  | **NA** | | **€0.091** | **€0.132** | **€0.091** | **€0.132** | **€0.091** | **€0.132** | **€0.091** | **€0.132** |  |
| **Technical CDR infrastructure (BECCS/biochar) — Bioeconomy pathway primarily** | | | | | | | | | | | | | |
| **BECCS: CO₂ capture, compression, transport & geological storage** | €100–2000/ t CO₂ capacity | Public | 0 | | 2.0 Mt / yr | | 2.0 Mt / yr | | 2.0 Mt / yr | | 5.1 Mt / yr | | Marcu & Varrichio (2025): <https://ercst.org/wp-content/uploads/2025/05/20250602-BECCS.pd>  Becidan & Hennig (2025): <https://task40.ieabioenergy.com/wp-content/uploads/sites/29/2025/05/IEA-Bioenergy_Intertask_BECCUS_Phase2_Oslo_WtE_final-report.pdf> |
|  |  |  | **NA** | | **€0.20** | **€4.00** | **€0.20** | **€4.00** | **€0.20** | **€4.00** | **€5.10** | **€10.20** |  |
| Amortised (20 yrs, 5% interest) | **€ bn/yr** |  | **NA** | | **€0.016** | **€0.321** | **€0.016** | **€0.321** | **€0.016** | **€0.321** | **€0.409** | **€0.818** |  |
| **Indicative total capital investment to 2050 (excluding recurring operational costs)** | | | | | | | | | | | | | |
| **TOTAL** |  |  | **€1.68** | **€1.83** | **€5.74** | **€12.06** | **€5.67** | **€11.92** | **€5.72** | **€12.02** | **€11.44** | **€19.45** |  |
| AMORTISED |  |  | **€1.51** | **€1.52** | **€1.82** | **€2.32** | **€1.82** | **€2.32** | **€1.82** | **€2.32** | **€2.28** | **€2.92** |  |
| Farmer median |  |  | €1.49 |  | €1.75 |  | €1.64 |  | €1.72 |  | €1.83 |  |  |
| Exchequer media |  |  | €0.02 |  | €0.47 |  | €0.47 |  | €0.47 |  | €0.92 |  |  |
| Overall median |  |  | €1.51 |  | €2.07 |  | €2.07 |  | €2.07 |  | €2.60 |  |  |

**Table S4 (see Supplementary Data Excel file).** Summary of output volumes and simple estimates of gross output values, subsidies and possible internalisation of external costs (GHG emission exceedance vs climate neutrality target (split gas), ammonia emissions to air and nutrient losses to water) at 2025 prices for the main products from Ireland's agriculture, forestry and other land use sector in 2020, and in 2050 under business as usual and the studied four climate-neutral transition pathways (split gas definition). Default results are presented first for a “least change” Shared Socioeconomic Pathway (SSP2) 2050 future context (Table S4a). Sensitivity analyses are applied by indicative (highly tentative) variations in subsidy distributions, input prices, output prices and/or volumes and internalisation of external costs: for SSP1 (Table S4b); SSP3 (Table S4c); SSP4 (Table S4d), and; SSP5 (Table S4c).

**Table S5 (see Supplementary Data Excel file).** Matrix of risk scores across nine categories (described in Table 5 main manuscript) across impacts associated with business as usual and the four studied climate-neutral transition pathways under five alternative future contexts as downscaled from the Shared Socioeconomic Pathways 1-5, via a series of inferred implications for Ireland (Table 3 in main manuscript). Colour coding is used to indicate low (green), medium (orange) and high (red) risks associated with each category, for each pathway in each SSP context. This is translated into scores of 1 (high risk) to 3 (low risk), aggregated using various weightings (to the right of main matrix). Results summarised in Figure 6 of the main manuscript. The risk matrix draws upon information from key biophysical performance indicators (Table S2), investment costs (Table S3) and indicative economic screening (Table S4).

**Figure S1.** Relative change in protein output and cattle numbers for the Sustainable Intensification pathway by 2050 compared with 2020 (set at 1), with different definitions of climate neutrality (GWP100 net zero, split gas, a simple downscaling of the EU net zero target involving a 30% reduction in agricultural emissions relative to 2020, and application of a GWP* CO_2_ warming equivalent net zero). The effect of excluding large but uncertain emissions (of carbon dioxide and methane) from rewetted and remaining-drained peat soils is also shown.

**Supplementary Data tabs S1-S5:** These 13 tabs present full greenhouse gas flux time series from 2020 to 2100, disaggregated by major greenhouse gas – carbon dioxide (CO_2_), methane (CH_4_) and nitrous oxide (N_2_O) – and by major source, including agriculture, existing forest (column F) and new forest from afforestation (column G), other land use (primarily organic soils), avoided emissions through product substitution with wood (column T; indicative only, not included in net zero calculations), land use change (column U), harvested wood products (HWP; column V), and carbon capture & storage (CCS) from wood (column W) and biogas (column X). Tabs S1 to S5 represent, respectively, business as usual (BAU) and the four climate-neutral pathways – sustainable intensification (SI), cattle plus nature (CN), protein diversification (PD) and bioenergy (BE). Climate-neutral pathway tabs include three derivatives: non-greenhouse gas constrained pathways (tabs “a”); pathways that comply with all-gas net zero (NZ) greenhouse gas emissions by 2050 (tabs “b”); pathways that comply with a split gas (SG) definition of climate neutrality by 2050 (tabs “c”).
